# Exploring the mechanism of Yixinyin for myocardial infarction by weighted co-expression network and molecular docking

**DOI:** 10.1038/s41598-021-01691-8

**Published:** 2021-11-19

**Authors:** Mengqi Huo, Lina Ma, Guoguo Liu

**Affiliations:** 1 Department of Cardiology, Liuzhou Traditional Chinese Medicine Hospital, Liuzhou, China; 2 School of Chinese Material Medica, Beijing University of Chinese Medicine, Beijing, China; 3 Rehabilitation Teaching and Research Section, Henan Medical College, Zhengzhou, China

**Keywords:** Gene regulatory networks, Genome informatics, Virtual drug screening

## Abstract

Yixinyin, the traditional Chinese medicine, has the effects of replenishing righteous qi, and promoting blood circulation to eliminate blood stagnation. It is often used to treat patients with acute myocardial infarction (MI). The purpose of our study is to explore the key components and targets of Yixinyin in the treatment of MI. In this study, we analyzed gene expression data and clinical information from 248 samples of MI patients with the GSE34198, GSE29111 and GSE66360 data sets. By constructing a weighted gene co-expression network, gene modules related to myocardial infarction are obtained. These modules can be mapped in Yixinyin PPI network. By integrating differential genes of healthy/MI and unstable angina/MI, key targets of Yixinyin for the treatment of myocardial infarction were screened. We validated the key objectives with external data sets. GSEA analysis is used to identify the biological processes involved in key targets. Through molecular docking screening, active components that can combine with key targets in Yixinyin were obtained. In the treatment of myocardial infarction, we have obtained key targets of Yixinyin, which are ALDH2, C5AR1, FOS, IL1B, TLR2, TXNRD1. External data sets prove that they behave differently in the healthy and MI (P < 0.05). GSEA enrichment analysis revealed that they are mainly involved in pathways associated with myocardial infarction, such as viral myocarditis, VEGF signaling pathway and type I diabetes mellitus. The docking results showed that the components that can be combined with key targets in YixinYin are Supraene, Prostaglandin B1, isomucronulatol-7,2′-di-O-glucosiole, angusifolin B, Linolenic acid ethyl ester, and Mandenol. For that matter, they may be active ingredients of Yixinyin in treating MI. These findings provide a basis for the preliminary research of myocardial infarction therapy in traditional Chinese medicine and provide ideas for the design of related drugs.

## Introduction

Myocardial infarction (MI) is one of the most common diseases in clinical practice, and the incidence rate has increased significantly in recent years^1^. Plaque rupture and subsequent thrombosis is the main cause of symptoms^2^. Factors that contribute to plaque vulnerability include the size of the lipid core, the thickness and cell structure of the fibrous cap, and the severity of the inflammatory response. Excessive inflammation may aggravate heart remodeling and cause heart failure ^3^. The arterial wall response to injury can be induced by a variety of mechanisms, including infection, reactive oxygen species (ROS), oxidized low-density lipoprotein, and hemodynamic shear stress. In addition, smoking, diabetes, hypercholesterolemia and hypertension are recognized risk factors related to the occurrence and development of myocardial infarction ^4^. Clinical methods such as drug thrombolysis and coronary stent implantation are commonly used to treat myocardial infarction, but they can relieve symptoms to a certain extent, but cannot repair necrotic myocardial tissue^5^.

Traditional Chinese medicine theory believes that Qi deficiency and blood stasis are the pathogenesis of patients with MI. Therefore, the treatment should be based on the adjustment of healthy Qi and the promotion of blood circulation. With the deepening of research on traditional Chinese medicine, more and more prescriptions are used for the treatment of patients with MI^6^. Yixinyin has the effect of promoting blood circulation and removing blood stasis, and is clinically used in the treatment of patients with MI^7^. Studies have shown that after patients are treated with Yixinyin, the plasma hsCRP, Fib and D-dimer levels are lower than those of the control group, and there is a significant difference in data between the groups (P < 0.05)^8^. Liu's research showed that chest tightness, chest pain score and serum TC, TG, LDL-C, apoB, and LP(a) levels of patients after treatment with Yixinyin were significantly lower than before treatment. The levels of HDL-C and apoA1 were significantly higher than before treatment. The differences were statistically significant (P < 0.05)^9^. The Salvia miltiorrhiza (Danshen) in the prescription can expand the coronary artery and resist platelet aggregation, thereby inhibiting the formation of thrombus^10^. Furthermoe, the Ligusticum chuanxiong hort (Chuanxiong) and Dalbergia odorifera (Jiangxiang) can effectively improve microcirculation and reduce blood viscosity^11,12^; Astragalus mongholicus(Huangqi), Pueraria lobata(Gegen), Pseudostellaria sylvatica (Taizishen) can promote blood circulation as well. It effectively reduces the body's inflammatory response, and restores the balance between the body's coagulation system and fibrinolytic system^13–15^. In addition, the prescription also contains Ophiopogon japonicus (Maidong), fried Glycyrrhiza uralensis Fisch (Zhigancao), Allium macrostemon Bunge. (Xiebai), Schisandra chinensis (Turcz.) Baill. (Wuweizi) and Shell of Trichosanthes kirilowii (Gualoupi). Unlike the synthetic drugs, which generally produce their efficacy based on the theory of one drug and one target, the efficacy of TCMs relies on the comprehensive effects of multiple compounds, multiple targets, and multiple pathways^16^. Therefore, it is difficult to discover the key components and targets of Yixinyin in the treatment of MI from the molecular level.

In recent years, researchers have studied the pathogenesis of MI in order to find more available treatments^16^. Network pharmacology is a commonly-used technology for analyzing the mechanism of traditional Chinese medicine from the compound molecules and targets^17^. In human biological networks, genes in the same pathway will manifest a tendency of co-expression. This also suggests that these genes may have similar biological functions. The weighted gene co-expression network is a collection of genes with these similar functions into modules^18^. With this feature, gene functions can be annotated and the biological processes involved in the module can be further revealed. This is of great significance to the study of the mechanism of Yixinyin in the treatment of MI.

In previous studies, PPI networks were often divided into modules based on the heterogeneity of the scale-free network^19,20^. These methods allow researchers to get more tightly connected modules in the network topology. However, in practical applications, we prefer to divide modules with tighter connections in biological functions^21^. The weighted gene co-expression network can modularize genes with similar functions and link them to clinical sample information^22^. As a result, we screened the genetic modules closely related to the clinical information in patients with MI, and combined these modules with the PPI network of Yixinyin. It preserves the biological significance of the module and removes redundant data from the PPI network, thus making the results more accurate^23^.

**Figure 1:**
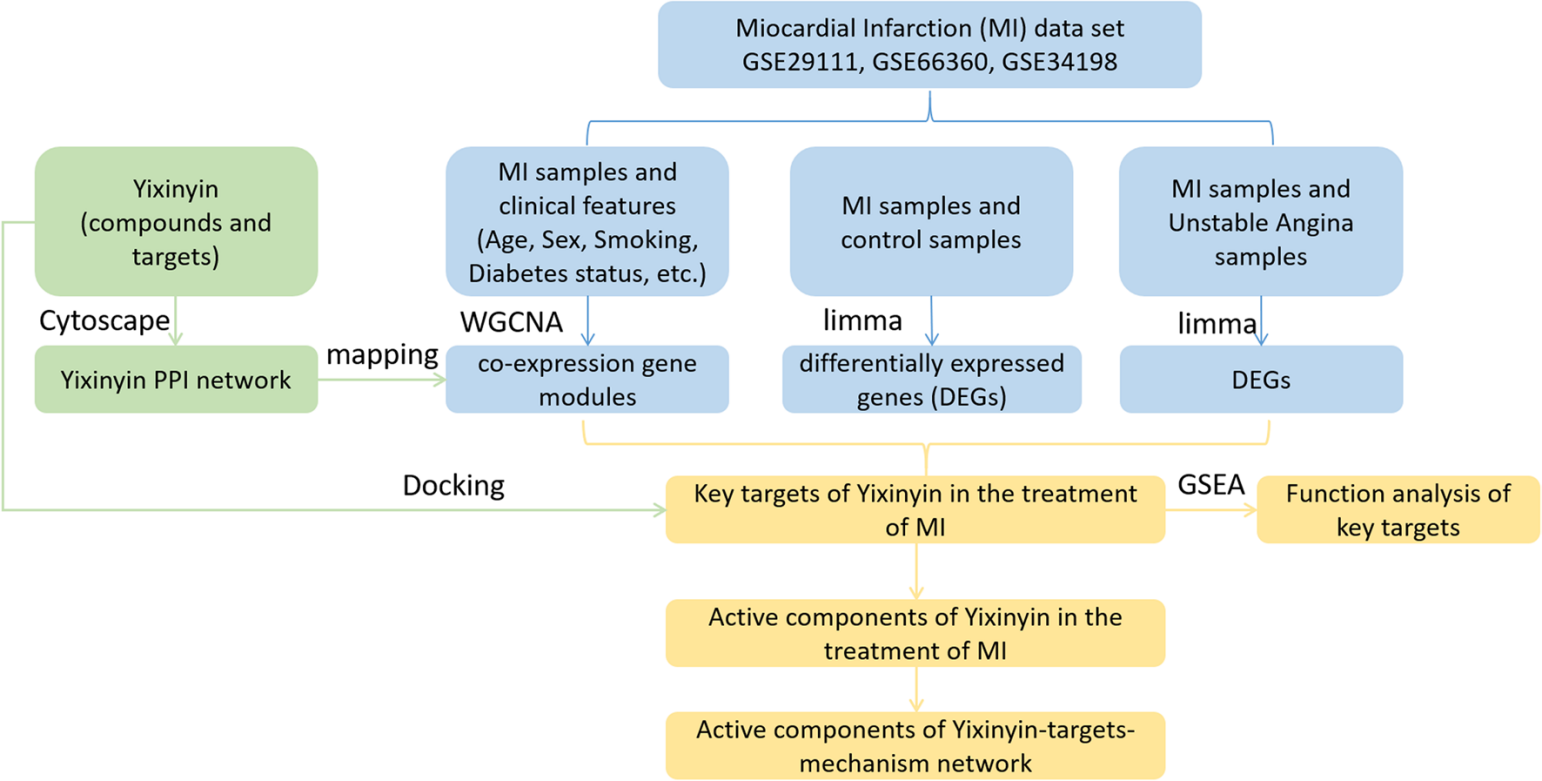
The flow chart of the whole research process.

In this study, we constructed a weighted gene co-expression network of MI samples based on the GSE34198, GSE29111 and GSE66360 data sets. Key gene modules closely related to clinical information are screened. Afterward, they are mapped to Yixinyin PPI network. By integrating the differential genes of healthy/MI patients and unstable angina (UA)/MI, key targets of Yixinyin for the treatment of MI were screened. In addition, these genes were confirmed to be differentially expressed in the GSE97320 data set. The main active substances in Yixinyin were obtained by docking. These findings may provide a new perspective for Yixinyin's treatment of MI. The flow chart of the whole research process is shown in Fig. 1.

## Results

### Acquisition of MI samples

The gene expression profiles of three data sets were downloaded from the GEO database. GSE34198, GSE29111, and GSE66360 contain a total of 248 samples and 33810 genes. Among them, GSE34198 included 49 MI and 48 control samples; GSE29111 contains 36 UA and 16 MI samples; GSE66360 contains 49 MI and 50 control samples.

### Identification of co-expression gene modules

**Figure 2:**
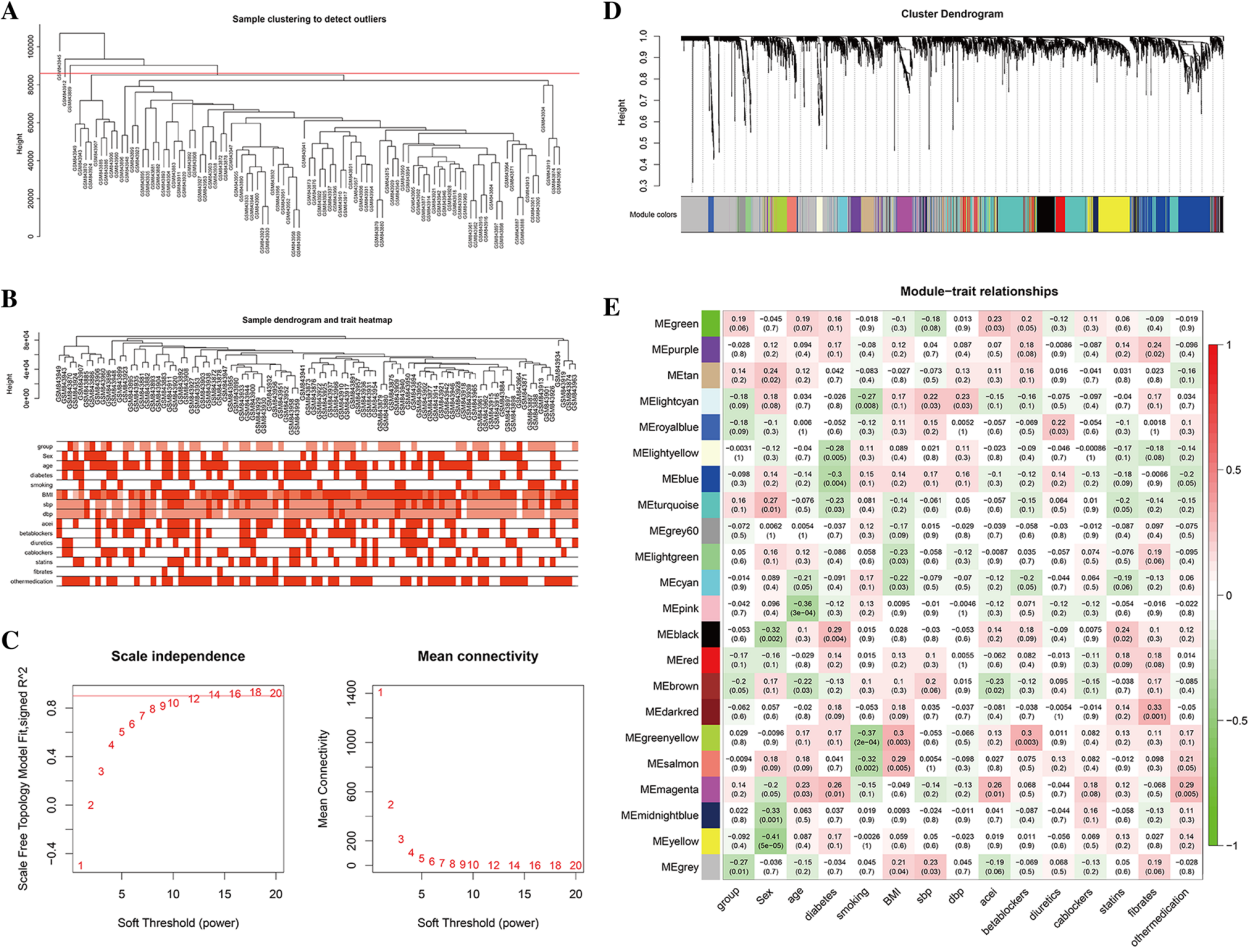
Drawing weighted gene co expression network of MI based on WGCNA package of R 3.5.1 software (https://www.r-project.org/). (**A**) Three outliers are removed from the hierarchical clustering graph. (**B**) Hierarchical clustering graph of samples. The orange in the figure represents the phenotype, and the color depth represents the value of the item. (**C**) Analysis of the scale-free fit index for various soft-thresholding powers (β), 12 was the most fit power value. (**D**) The cluster dendrogram of genes in GSE34198. Each branch in the figure represents one gene, and every color below represents one co-expression module. (**E**) Heat map of the relationship between the characteristic genes of modules and MI. Correlation coefficient along with P value in parenthesis underneath. Color-coded according to correlation coefficient (legend at right).

There are 97 samples in the GSE34198 data set. The data were combined and converted into expression matrix that contain 24,612 genes. A total of 2264 genes in the top 25% of variance were selected for WGCNA. The clinical phenotype data include gender, age, BMI, sbp, dbp, diabetes status, smoking status, acei, betablockers, diuretics, Ca blockers, statins, fibrates and other medication, as shown in Table S1 and Fig. 2B. We identify outliers based on the distance of sample clustering. The cutHeight is set to 85,000 to eliminate three obvious outliers (GSM843945, GSM843912, GSM843889) (Fig. 2A). Subsequently, the remaining 94 samples were background-corrected, standardized and polymerized to obtain a gene expression matrix. The soft threshold should be the smallest integer when the fitting coefficient R2 reaches 0.9, so that the constructed gene co-expression network can conform to the characteristics of the scale-free network. We choose β=12 as the soft threshold for this study (Fig. 2C). The dynamic tree cutting algorithm and the hclust function merge highly similar modules, and finally a clustering dendrogram can be obtained (Fig. 2D).

A total of 21 gene modules were identified in this study. The gray modules are gene sets that cannot be collected in other modules. The correlation between these modules and the phenotype (gender, age, BMI, sbp, dbp, diabetes status, and so on) is calculated based on the characteristic vectors of each module, as shown in Fig. 2E. The heat map illustrates that Black, midnightblue, and yellow are significantly negatively correlated with gender (cor = − 0.32, P = 0.002, cor = − 0.33, P = 0.001, cor = − 0.41, P < 0.001). Pink has a significant negative correlation with age (cor = − 0.36, P < 0.001). Blue was significantly negatively correlated with diabetes status (cor = − 0.30, P = 0.004). Greenyellow and salmon were significantly negatively correlated with smoking (cor = − 0.37, P < 0.001, cor = − 0.32, P = 0.002), however, they were significantly positively correlated with BMI (cor = 0.30, P = 0.003, cor = 0.29, P = 0.005). Greenyellow was significantly positively correlated with betablockers (cor = 0.30, P = 0.003). Darkred was significantly positively correlated with fibrates (cor = 0.33, P = 0.001).

**Figure 3:**
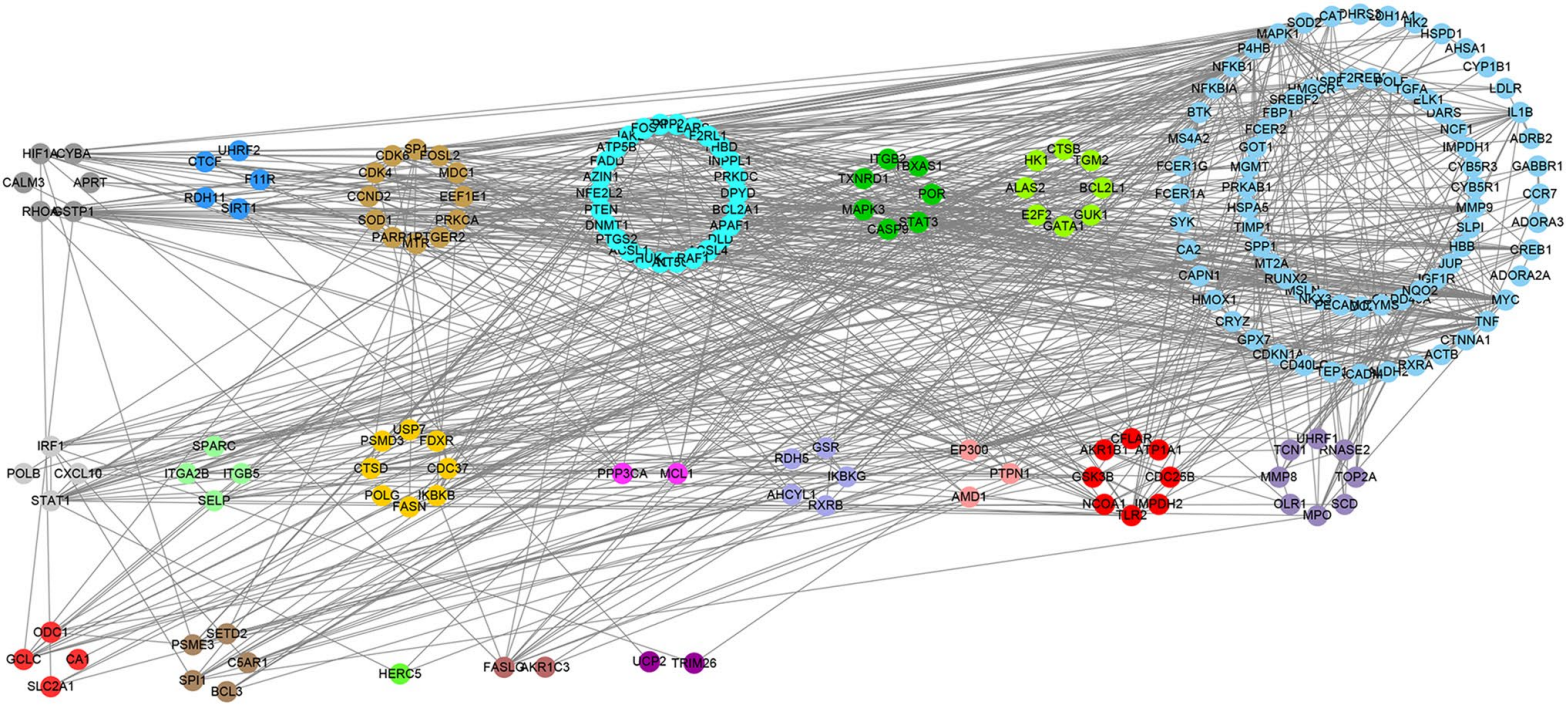
Constructing network based on Cytoscape 3.7.0. (https://cytoscape.org/). Module diagram of MI weighted gene co-expression network mapped to Yixinyin PPI network. The different colors in the figure represent the corresponding modules. The black line represents the PPI relationship between the nodes.

A total of 169 component molecules and 717 Yixinyin targets were obtained from the Chinese medicine database. Afterwards, the PPI between the targets is imported into the Cytoscape to build Yixin's PPI network. The network contains 581 nodes and 4137 edges, as shown in Fig. S1. The figure is drawn by cytoscpe 3.7.0 software (https://cytoscape.org/). The weighted gene co-expression network of MI is mapped to the Yixinyin PPI network, and the PPI network of Yixinyin is obtained for MI treatment. It contains a total of 64 nodes and 249 edges (Fig. 3).

### Analysis of differentially expressed genes

**Figure 4:**
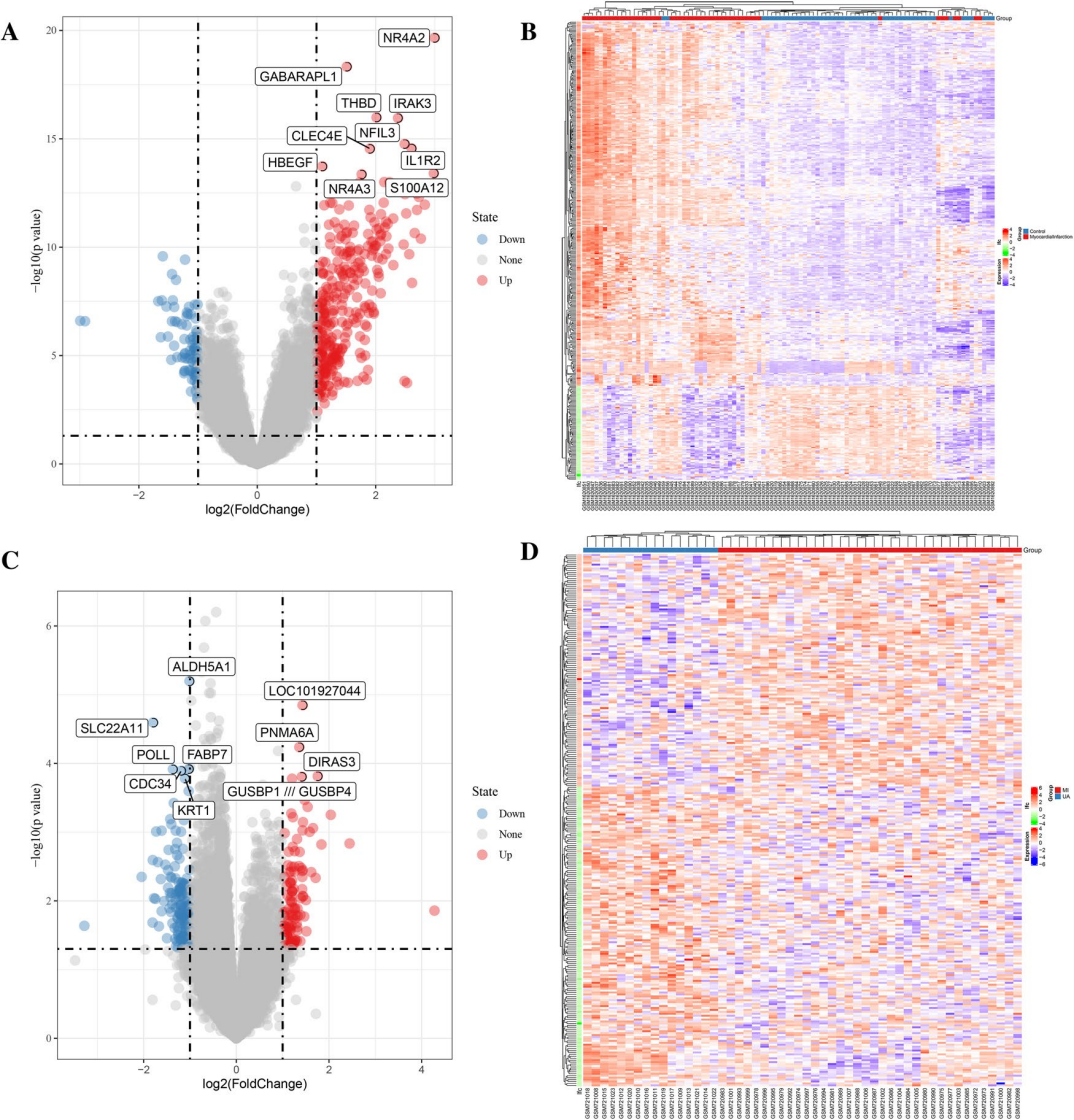
DEGs identified based on GEO database. This figure is drawn by the sangerbox platform (http://sangerbox.com/Tool). (**A**) In the GSE66360 data set, the volcano map of mRNA expression values between MI samples and control samples. Red is up-regulated genes, and blue is down-regulated genes. (**B**) In the GSE66360 data set, hierarchical clustering heat map of DEGs. (**C**) In the GSE29111 data set, the volcano map of mRNA expression values between MI samples and UA samples. Red is up-regulated genes, and blue is down-regulated genes. (**D**) In the GSE29111 data set, hierarchical clustering heat map of DEGs.

A total of 390 DEGs were obtained in the GSE66360 data set. Compared with control group samples, there are 310 mRNA expressions in MI samples illustrated an up-regulation trend, and 80 mRNA expressions show a down-regulation trend (Fig. 4A,B). Besides, a total of 277 DEGs were screened out in the GSE29111 data set. Compared with the UA group, 121 mRNA expressions of MI samples presented an upward-regulated trend, and 156 mRNA expressions showed a downward-regulated trend (Fig. 4C,D). This figure is drawn by the sangerbox platform (http://sangerbox.com/Tool^24^. We have drawn volcano maps of the two datasets respectively. Genes and samples with the same or similar expression behavior are gathered and drawn into heat maps.

**Figure 5:**
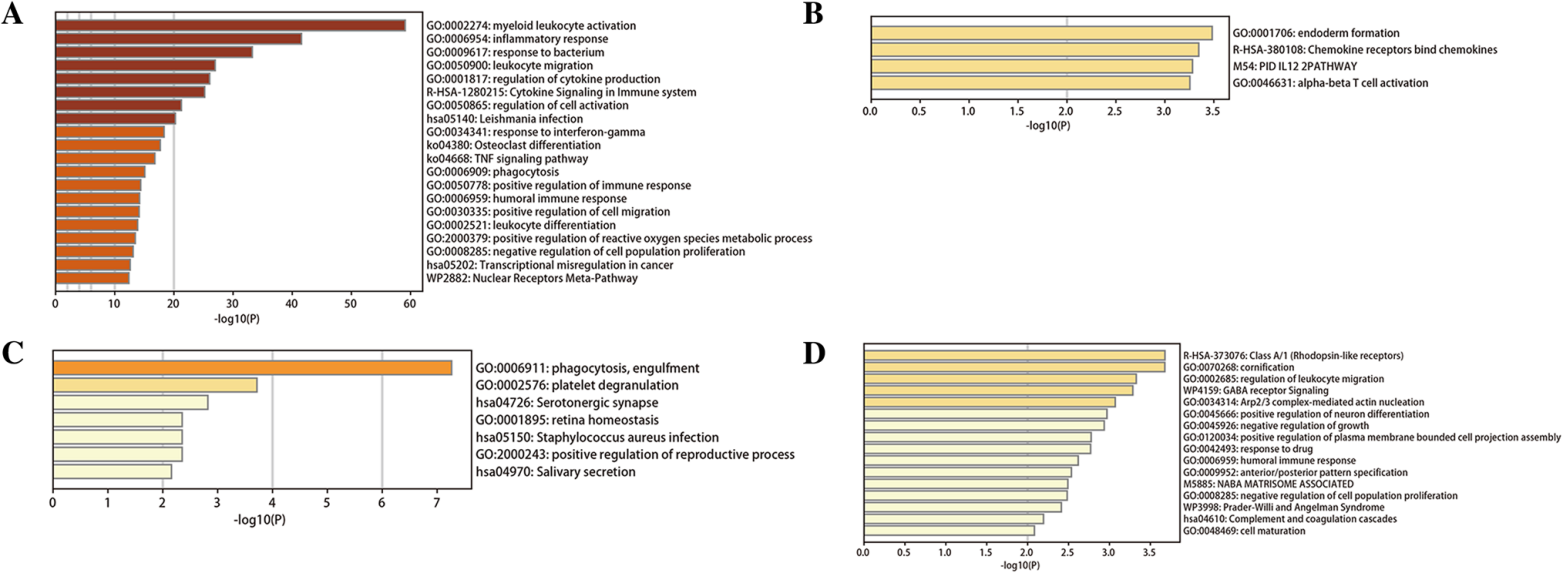
Enrichment analysis of DEGs based on metascape database (https://metascape.org/gp/index.html#/main/step1). (**A**) Enrichment analysis results of down regulated genes in GSE66360. (**B**) Enrichment analysis results of up regulated genes in GSE66360. (**C**) Enrichment analysis results of up regulated genes in GSE29111. (**D**) Enrichment analysis results of down regulated genes in GSE29111.

DEGs in two datasets were analyzed by metascape. The results are shown in Figure 5. Up regulated genes in GSE66360 are significantly enriched in myeloid leukocyte activation, inflammatory response, response to bacterium, leukocyte migration, regulation of cytokine production. Down regulated genes in GSE66360 are significantly enriched in endoderm formation, chemokine receptors bind chemokines, PID IL12 2PATHWAY, alpha-beta T cell activation. Up regulated genes in GSE29111 are significantly enriched in phagocytosis, engulfment, platelet degranulation, Serotonergic synapse, retina homeostasis, positive regulation of reproductive process. Down regulated genes in GSE29111 are significantly enriched in Class A/1 (Rhodopsin-like receptors), cornification, regulation of leukocyte migration, GABA receptor Signaling, Arp2/3 complex-mediated actin nucleation. The detailed results are shown in Table S2.

### Identification of key genes

**Figure 6:**
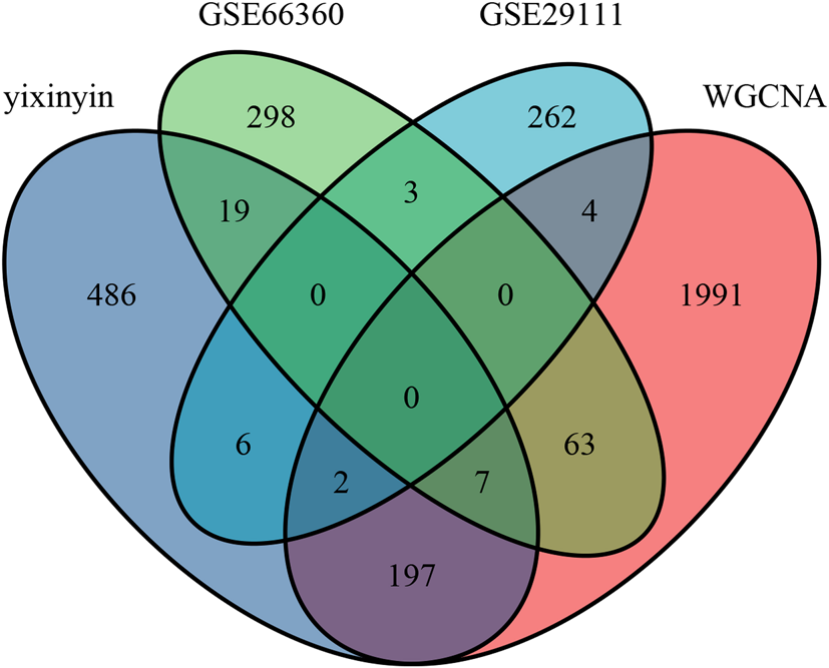
Venn diagram of genes screened based on GSE34198, GSE29111, Yixinyin PPI and WGCNA.

We take the mapped PPI network module with the intersection of the differential genes of GSE29111 and GSE66360. The genes in the cross-region are selected as key genes. As shown in Fig. 6, the above procedure is drawn as a Venn diagram. There are 7 genes in the intersection between the mapped network module and GSE66360, which are ALDH2, IL1B, TLR2, C5AR1, FOS, THBD and ACSL1. There are 2 genes in the intersection between the mapped network module and GSE29111, which are E2F2 and TXNRD1. Therefore, these genes in the cross-region are considered as key genes.

### Verification of key genes based on external data sets

**Figure 7:**
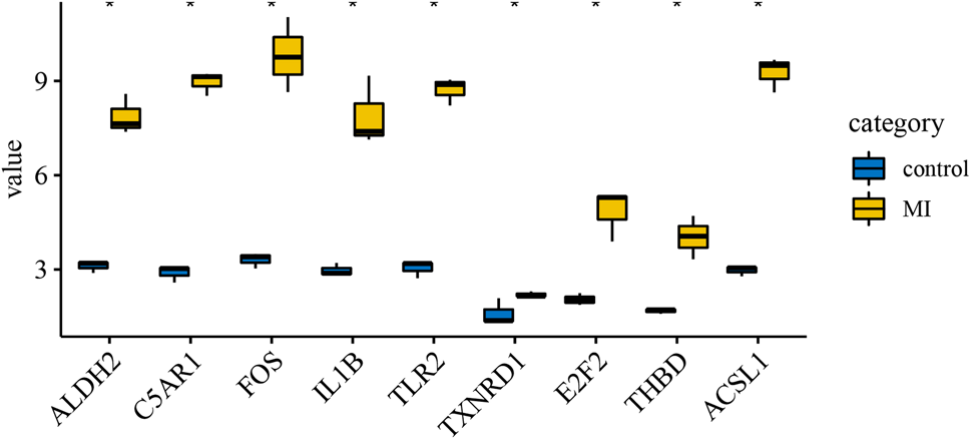
Box plot of expression values of ALDH2, IL1B, TLR2, C5AR1, FOS, THBD, ACSL1, E2F2, TXNRD1 in MI and control samples. *Represents P < 0.05. This figure is drawn by the sangerbox platform (http://sangerbox.com/Tool).

We downloaded the GSE97320 dataset in the GEO database to verify key genes. As shown in Fig. 7, the expressions of ALDH2, IL1B, TLR2, C5AR1, FOS, THBD, ACSL1, E2F2, and TXNRD1 in MI samples were significantly higher than those in control samples. This is consistent with the results we analyzed in the GSE34198, GSE29111 and GSE66360 data sets.

### GSEA analysis of key genes

**Figure 8:**
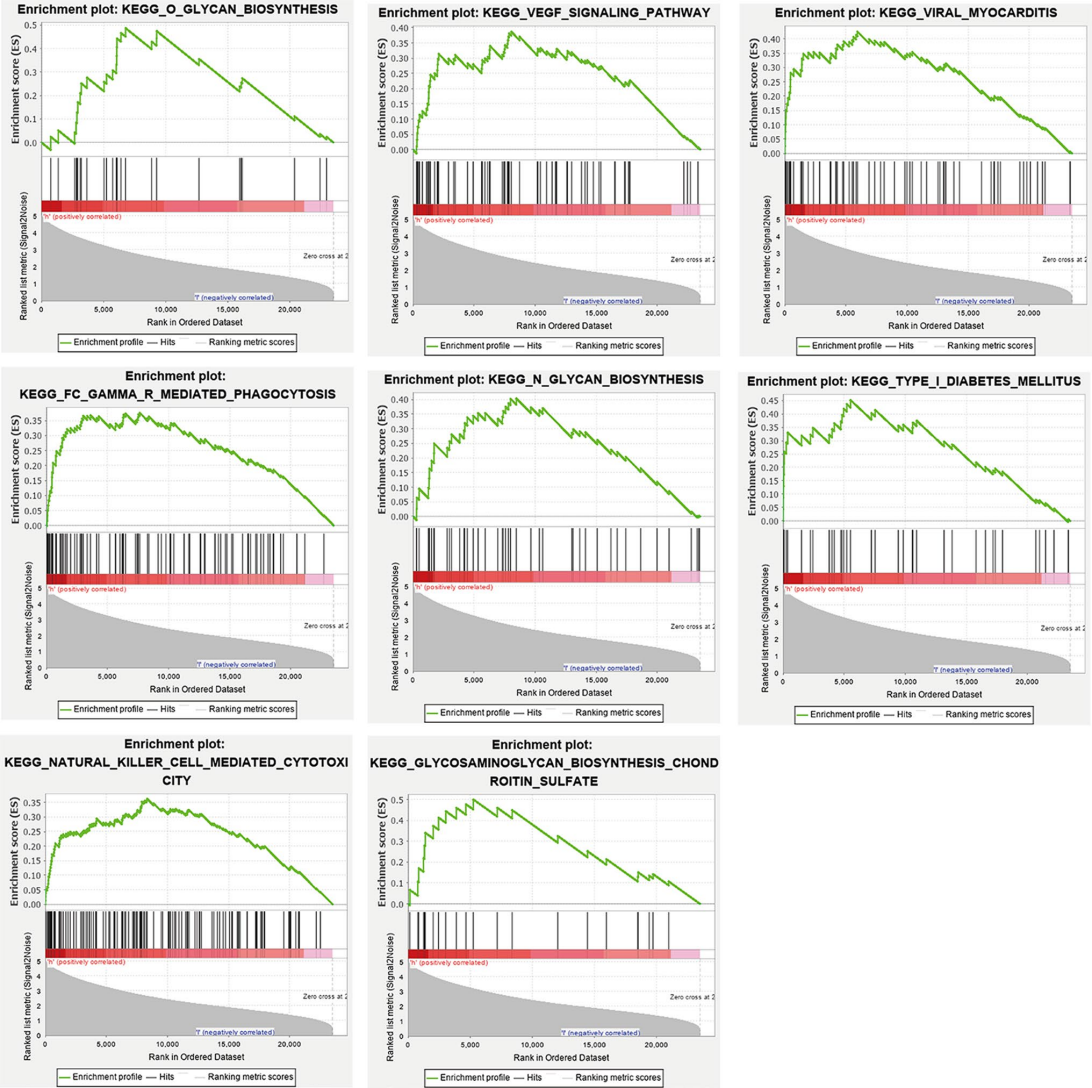
Enrichment analysis of key genes in high expression group based on GSEA 4.0.3 software (http://software.broadinstitute.org/gsea/downloads.jsp).

We divided the samples into high expression groups and low expression groups based on the expression of key genes. Key genes are up-regulated in GSE29111, GSE66360, and GSE97320. Therefore, we used GSEA analysis to obtain pathways related to the high expression group. There are 8 pathways regulated by the high expression group of key genes. They are viral myocarditis, type I diabetes mellitus, O glycan biosynthesis, VEGF signaling pathway, FC gamma r mediated phagocytosis, natural killer cell mediated cytotoxicity, N glycan biosynthesis, glycosaminoglycan biosynthesis chondroitin sulfate (Fig. 8).

### Identification of potentially active compounds

**Figure 9:**
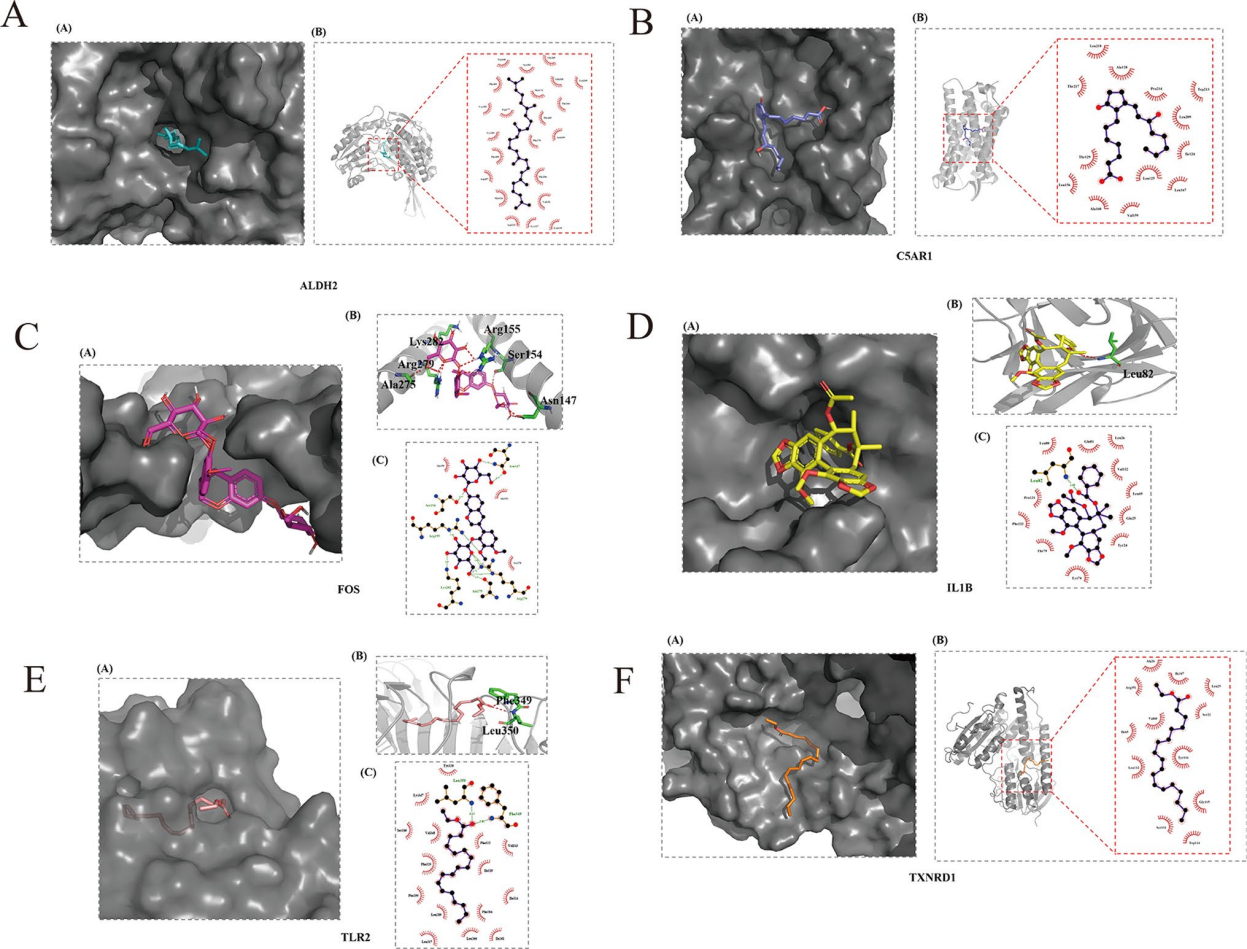
The complex model of ligand and receptor as well as their interactions. The figure is drawn using the SYBYL-X 2.0 software (Tripos, St. Louis, MO). (**A**) The best combination with ALDH2 is Supraene. (**B**) The best combination with C5AR1 is prostaglandin B1. (**C**) The best combination with c-Fos is isomucronulatol-7,2'-di-O-glucosiole. (**D**) The best combination with IL1 beta is angusifolin B. (**E**) The best combination with TLR2 is Linolenic acid ethyl ester. (**F**) The best combination with TRXR1 is Mandenol.

A total of 169 astragalus components were obtained through the TCMSP database, and the component information is shown in Table S3. Only the proteins corresponding to ALDH2, C5AR1, IL1B, FOS, TLR2, and TXNRD1 have crystal structures that can be used, so we selected the above 6 targets for molecular docking. The crystal structures with PDB codes 3INL, 5O9H, 5R88, 1FOS, 2Z7X, 2J3N were selected as the docking models of ALDH2, C5AR1, IL1B, FOS, TLR2, TXNRD1(Table 1 and Fig. 9A–F). The figure is drawn using the SYBYL-X 2.0 software (Tripos, St. Louis, MO)^25^. All components are sequentially docked with ALDH2, C5AR1, IL1B, FOS, TLR2 and TXNRD1 to obtain the corresponding binding free energy. The docking results of all compounds and targets are shown in Table S4.

**Table 1:** Protein crystal information and the score of the best binding compounds.

Key genes	Receptor	PDB ID	Ligand	Total score
ALDH2	ALDH2	3INL	MOL001506	12.1100
C5AR1	C5AR1	5O9H	MOL007651	8.1762
IL1B	IL1 beta	5R88	MOL009235	6.4750
FOS	c-FOS	1FOS	MOL000439	7.6386
TLR2	TLR2	2Z7X	MOL007197	11.0697
TXNRD1	TRXR1	2J3N	MOL001494	5.2476

### Mechanism network of Yixinyin in treating MI

**Figure 10:**
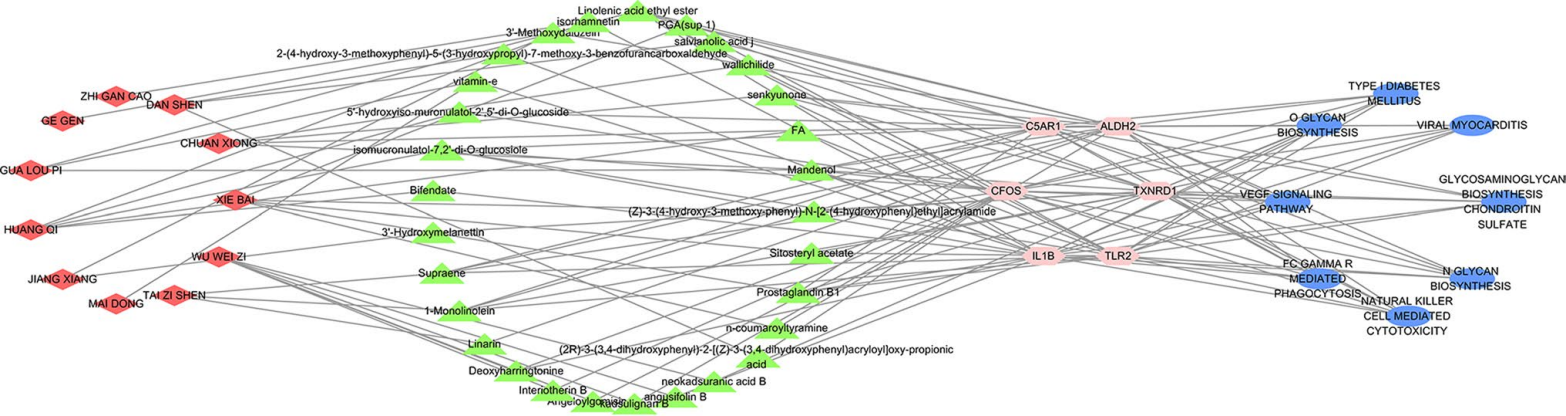
Yixinyin-component-target-pathway network diagram. The figure is drawn by cytoscpe 3.7.0 software (https://cytoscape.org/). The red node represents the herb in Yixinyin. The green node represents the compounds in Yixinyin. The pink node represents the key gene. The blue node represents the pathway they participate in.

As shown in Fig. 10, for each target, we only selected the top ten components, and drew the Yixinyin-component-target-pathway network diagram.The figure is drawn by cytoscpe 3.7.0 software (https://cytoscape.org/). As shown in Fig. 9A–F, we show the interaction between the compound with the highest score and the target. The best combination with ALDH2 is Supraene. The best combination with C5AR1 is prostaglandin B1. The best combination with c-Fos is isomucronulatol-7,2′-di-O-glucosiole. The best combination with IL1 beta is angusifolin B. The best combination with TLR2 is Linolenic acid ethyl ester. The best combination with TRXR1 is Mandenol. There are also some ingredients that have good binding effects with multiple targets, such as senkyunone, isomucronulatol-7, 2'-di-O-glucosiole, Supraene, 1-Monolinolein, Linolenic acid ethyl ester, Ethyl linoleate. According to the network diagram, we found that every Chinese medicine in Yixinyin has active components that bind to key targets. This result shows that the ingredients in the prescription are synergistic and participate in the treatment of MI.

## Discussion

In this study, we analyzed the gene expression data of 248 MI and control samples. Throughout the analysis of weighted gene co-expression network, 21 co-expressed gene modules were obtained. They are mapped in the PPI network to obtain the gene modules of Yixinyin to treat MI. By the integration of the DEGs of healthy/MI and unstable angina/MI, the potential targets of Yixinyin for the treatment of MI (ALDH2, C5AR1, CFOS, IL1B, TLR2, TRXR1) have been obtained. External data sets have also verified the significance of these targets. GSEA analysis indicates that the high expression groups of ALDH2, C5AR1, CFOS, IL1B, TLR2, TRXR1 jointly participate in the pathways of viral myocarditis, VEGF signaling pathway and type I diabetes mellitus associated with MI.

Toll-like receptor-2 (TLR2) is an essential protein molecule involved in non-specific immunity. It is apparent drawn from studies that by activating the nuclear factor κB and up-regulating interleukin-1β (IL1B), TLR2 can induce the proliferation of cardiomyocyte hypertrophy, fibroblasts and vascular endothelial cell proliferation. MiR-499 and other miRNAs are involved in ischemic myocardial protection. This protection may be achieved by the inhibition of TLR2 and the reduction of inflammatory cytokine release (including IL-1β and IL-6). These effects eventually lead to a dropping in the area of MI^26^. Therefore, regulating TLR2 signal may provide a new treatment strategy for heart failure. Mitochondrial aldehyde dehydrogenase (ALDH2) is a key enzyme that protects the heart during ischemia and reperfusion. Experiments have shown that both protein kinase C ε (PKCε) agonists and ethanol can increase ALDH2 during cardiac ischemia in mice. Therefore, its high expression in patients with MI may be a protective effect of the human body^27^. The activation of ALDH2 enzyme can reduce the differentiation of cardiac fibroblasts. This may be a promising strategy to alleviate myocardial fibrosis, and thus develop the cardioprotective drugs like ALDH2 activators^28^. Alda-1 is the first ALDH2 activator discovered, and its cardioprotective effect is widely recognized in vivo^29^. FOS is the coding gene of c-fos. After the coronary artery conjunctiva in rats, the expression of miR-101a/b in the peri-infarct area decreased. c-Fos was found to be the target of miR-101a. After silencing c-Fos, cardiac function has improved notably^30^. Some clinical trials have shown that postprandial hyperglycemia makes a difference in the occurrence and development of MI. Hyperglycemia can induce c-fos gene expression. For that matter, inhibiting the expression of c-fos may become a new treatment strategy for MI^31,32^. Studies have shown that in the absence of C5aR1, the levels of IL-1 beta and IL-6 decreased in the plasma of mice with cardiac insufficiency are reduced. IL-1 beta and IL-6 are closely related to the development of MI^33^. Hence, inhibiting the expression of C5aR1 is conducive to the reduction of the risk of MI. There has been no report about Yixinyin treating MI through these targets.

We discovered some potential active compounds in Yixinyin through docking. Studies have shown that squalene has a protective effect on rats with MI induced by isoproterenol. Its protective effect on the heart is attributed to its ability to reduce the release of hydrolase or to strengthen the myocardial membrane through its antioxidant properties against free radicals^34^. Linolenic acid ethyl ester is a derivative of linolenic acid and has similar physiological effects to linolenic acid. Studies have shown that linolenic acid is associated with a lower risk of acute myocardial infarction. Eating vegetable oils rich in linolenic acid can have an important protective effect on the cardiovascular system^35^. Ethyl linoleate is a derivative of linoleic acid and has similar physiological effects to linoleic acid. The combined administration of linoleic acid and nitrite can play a cardioprotective effect in the event of MI. It can reduce the level of hydrogen peroxide and significantly change the activity of myocardial mitochondrial respiration and electron transport chain^36^. It was found that the 1-Monolinolein may be the key compound for the hemostatic effect of Cortex Moutan. It is involved in related pathways of complement, coagulation cascade and platelet activation^37^. At present, there is no research on the compounds such as angusifolin B, isomucronulatol-7,2′-di-O-glucosiole, Mandenol, senkyunone in the treatment of MI. They may be potentially active compounds, which need to be further verified through pharmacological experiments.

In this study, we obtained gene modules related to MI by constructing a weighted gene co-expression network. As a result, the resulting modules have a better connection in biological functions. After that, we mapped the gene modules to the PPI network of Yixinyin, and screened out the key genes. These key genes are not only the potential targets of Yixinyin, but also closely related to MI. Compared with the direct construction of Yixinyin PPI network, this method can better integrate the transcriptome data of the disease, making the research results more concentrated^38^. Admittedly, our approach also has a certain limitation. Due to factors such as experimental cost and social ethics, the number of patient samples experienced in this study is very limited^39^. In addition, our research lacks the validation of animal or cell experiments. In future research, we will conduct perform pharmacodynamic analysis of potential ingredients, and biologically validate potential key genes through in vivo and in vitro experiments.

## Materials and methods

### Data download and preprocessing

In this study, in order to discover genes related to MI, we downloaded the GSE66360 data set containing samples of MI patients and healthy controls. In addition, recurrent unstable angina (UA; clinical symptoms of cardiac ischemia without myocardial necrosis) is a common development process of MI (clinical symptoms of cardiac ischemia with myocardial necrosis). Therefore, we also downloaded GSE29111 which contains samples of UA and MI. Further, in order to explore the relationship between clinical characteristics of MI patients and gene modules, we downloaded the GSE34198 data set containing complete clinical information. All three data sets come from the GEO database (https://www.ncbi.nlm.nih.gov/geo/^40^. The data need to be pre-processed prior to analysis, including the extraction of clinical information from samples based on the Illumina human-6 v2.0 expression beadchip and Affymetrix Human Genome U133 Plus 2.0 Array platforms, the construction of a gene expression matrix, and the conversion of probe names into gene names. The study population consisted of patients aged 18–80 years old of both sexes who presented to one of fve San Diego County medical centers with the diagnosis of acute myocardial infarction (AMI). Healthy control patients between the ages of 18 and 35 without a history of chronic disease and diseased control patients (with known but stable cardiovascular disease) of between the ages of 18–80 years old were recruited to outpatient clinical centers afliated with Te Scripps Translational Science Institute (STSI) through which Institutional Review Board (IRB) approval for all aspects of this study was obtained. All experiments were performed in accordance with relevant guidelines and regulations. Recruitment of all patients occurred from February 2008 through July 2014, and experiments were conducted with patient samples in phases as separate cohorts. Informed consent was obtained from all subjects in this study.

### Construction of weighted gene co-expression network

The GSE34198 data set with relatively complete clinical information was selected for weighted gene co-expression network analysis (WGCNA). The top 25% of the genes in the variance are used for subsequent calculations (i.e., the genes that have significantly changed in each sample are selected). The gene expression matrix performs the process of missing value (deleting genes with more missing values) and the elimination of outliers. The gene expression data of the remaining samples are used to construct a weighted gene co-expression network. According to the standard of the scaleless network, the appropriate weighting factor β is selected. The above process can be implemented by taking advantage of the pickSoftThreshold and softConnectivity function in the WGCNA package. The correlation between genes is calculated based on topological overlap (topological matrix, TOM). After that, a Dynamic Tree Cut is performed to divide the gene modules. The hclust function is used to merge highly similar modules for clustering dents. In this regard, the size of minimum module is set to 10 and a gene tree diagram is drawn^41^.

The gene module is then associated with the clinical feature, and the significance of the p value is calculated to obtain the gene significance (GS). It represents the correlation between genes and phenotypes. For each module, we define module membership (MM) as the correlation of module eigengene and the gene expression profile. We select modules that are highly correlated with the phenotypic characteristics of MI.

### PPI network of Yixinyin in the treatment of MI

The component data of Yixinyin stems from the Chinese Medicine System Pharmacology Database (TCMSP, http://ibts.hkbu.edu.hk/LSP/tcmsp.php^42^. The oral bioavailability (OB) and drug-like properties (DL) related to ADME are used to screen the active ingredients of Yixinyin. The screening criteria are: OB≥30% and DL≥0.18^43^. In addition, the target data of Yixinyin comes from the TCMSP database, the Encyclopedia of Traditional Chinese Medicine (ETCM, http://www.nrc.ac.cn:9090/ETCM/) and the BATMAN database (BATMAN, http://bionet.ncpsb.org/batman-tcm/)^44,45^. The PPI data between Yixinyin targets comes from the string database (https://string-db.org^46^. The PPI data with confidence level greater than 0.7 are imported into the Cytoscape 3.7.0 software (https://cytoscape.org/) to build the Yixinyin PPI network^47^.

Taking the PPI network of Yixinyin as the background, the gene modules obtained in the MI WGCNA are mapped into it. The Uniprot database (https://www.uniprot.org/) is used for the conversion of gene names and target names^48^. Therefore, the coexpression data based on WGCNA automatically divides the PPI network into multiple modules.

### key genes of Yixinyin in treating MI

In order to further clarify the genetic differences between patients with MI and the control group, we used the limma package in the R 3.5.0 software to analyze the differentially expressed genes for MI and the control group in the GSE66360 data set. Heat map and volcano map using sangerbox platform(http://sangerbox.com/Tool)^24^. The screening criterion is that |log2(fold change)| > 1 and P < 0.05 are differentially expressed genes (DEGs). Among them, log2(fold change) > 1 is marked as an up-regulated gene; log2(fold change) < − 1 is marked as a down-regulated gene. It is not the DEG that does not meet the above criteria. Similarly, we also screened the DEGs for UA and MI in the GSE29111 data set. In order to identify the biological functions of DEGs, this study mapped genes to the online website metascape (https://metascape.org/gp/index.html#/main/step1) for condensed analysis^49^. The entries with P<0.05 are considered statistically significant.

Based on the co-expression between genes, we divided the Yixinyin PPI network into modules. Specifically, the mapping gene module reflects the main PPI mechanism for the Yixinyin in treating MI. If nodes in the module network are differentially expressed between patients and healthy samples, or differentially expressed in UA and MI, they may be the primary regulatory targets of Yixinyin for the treatment of MI. Therefore, in order to screen for key genes, we take the intersection of the mapped network module and DEGs, where in the cross-region genes are selected as the key genes.

### Verification of key genes based on external data sets

We downloaded the GSE97320 dataset in the GEO database to verify key genes. The expression data of key genes are extracted to evaluate whether they are differentially expressed between MI and control group.

### GSEA analysis of key genes

In order to identify the biological processes involved in key genes at different expression levels, we divided the samples into high expression group and low expression group on the basis of expression values of key genes analyzed by GSEA Enrichment. The expression matrix of key genes in GSE97320 is used as an annotation file for sample grouping. Import the above files into GSEA 4.0.3 and select the c2.cp.kegg.v7.0.symbols.gmt data set in the msigdb database as the reference gene set. The cut off values of gene set enrichment are NOM p-val < 0.05 and FDR q-val < 0.25.

### Molecular docking

The 3D structure of all molecules in Yixinyin is downloaded from the PubChem database (https://pubchem.ncbi.nlm.nih.gov/^50^. The 3D structure of proteins corresponding to key genes is downloaded from the PDB protein database (http://www.rcsb.org/pdb/home/home.do^51^. We prefer a crystal structure that is compounded with the appropriate ligand and has a higher resolution^52^. The SYBYL-X 2.0 package (Tripos, St. Louis, MO) is used to connect Yixinyin ingredients and key proteins^25^. Before docking simulation, all ligands are removed from the protein-receptor complex and polar hydrogen atoms and charges are added. Finally, the compound is docked to the target by using a semi-flexible docking method.

## Conclusions

In this study, we analyzed the gene expression data from 248 MI and control samples, and screened and obtained the key genes of ALDH2, C5AR1, FOS, IL1B, TLR2, TXNRD1, which are also differentially expressed in GSE29111 and GSE66360. This result has been validated in an external data set. The ingredients in Yixinyin that can be combined with these targets are Supraene, Prostaglandin B1, isomucronulatol-7,2′-di-O-glucosiole, angusifolin B, Linolenic acid ethyl ester, and Mandenol, and they may be the active ingredients in Yixinyin. These findings provide a basis for the preliminary research in the treatment of MI with traditional Chinese medicine, as well as the crucial ideas for the design of related drugs.

## Supplementary Information

Supplementary Information.

## Data Availability

The datasets supporting the conclusions of this article are included within the article and its additional files.
