# Publisher Correction: Exploring the mechanism of Yixinyin for myocardial infarction by weighted co-expression network and molecular docking

**DOI:** 10.1038/s41598-021-03040-1

**Published:** 2021-12-21

**Authors:** Mengqi Huo, Lina Ma, Guoguo Liu

**Affiliations:** 1 Department of Cardiology, Liuzhou Traditional Chinese Medicine Hospital, Liuzhou, China; 2 School of Chinese Material Medica, Beijing University of Chinese Medicine, Beijing, China; 3 Rehabilitation Teaching and Research Section, Henan Medical College, Zhengzhou, China

Correction to: *Scientific Reports* 10.1038/s41598-021-01691-8, published online 19 November 2021

The original version of this Article contained an error in Affiliation 3, which was incorrectly given as ‘Rehabilitation Teaching and Research Section, Henan Medical College, Kaifeng, China’. The correct affiliation is listed below:

Rehabilitation Teaching and Research Section, Henan Medical College, Zhengzhou, China.

The original Article has been corrected.

