# Supplementary material for: Exploring the mechanism of Yixinyin for myocardial infarction by weighted co-expression network and molecular docking
Source: Sci Rep. 2021 Nov 19;11:22567. doi: 10.1038/s41598-021-01691-8 (PMC8604969; doi:10.1038/s41598-021-01691-8)
Supplement: Supplementary file 1 — Supplementary Information. [file 41598_2021_1691_MOESM1_ESM.docx]

Supplementary Data for

Exploring the mechanism of Yixinyin for myocardial infarction by weighted co-expression network and molecular docking

Mengqi Huo ^2#^, Lina Ma ^3#^ and Guoguo Liu ^1,^*

^1^ Department of Cardiology, Liuzhou Traditional Chinese Medicine Hospital; 512096387@qq.com

^2^ School of Chinese Material Medica, Beijing University of Chinese Medicine; chouchangkehuo@126.com

^3^ Rehabilitation teaching and research section, Henan Medical College; 2008050087@hamc.edu.cn

* Correspondence: 512096387@qq.com; Tel.: (8613737279631)

^#^ Mengqi Huo and Lina Ma contributes equally to this work


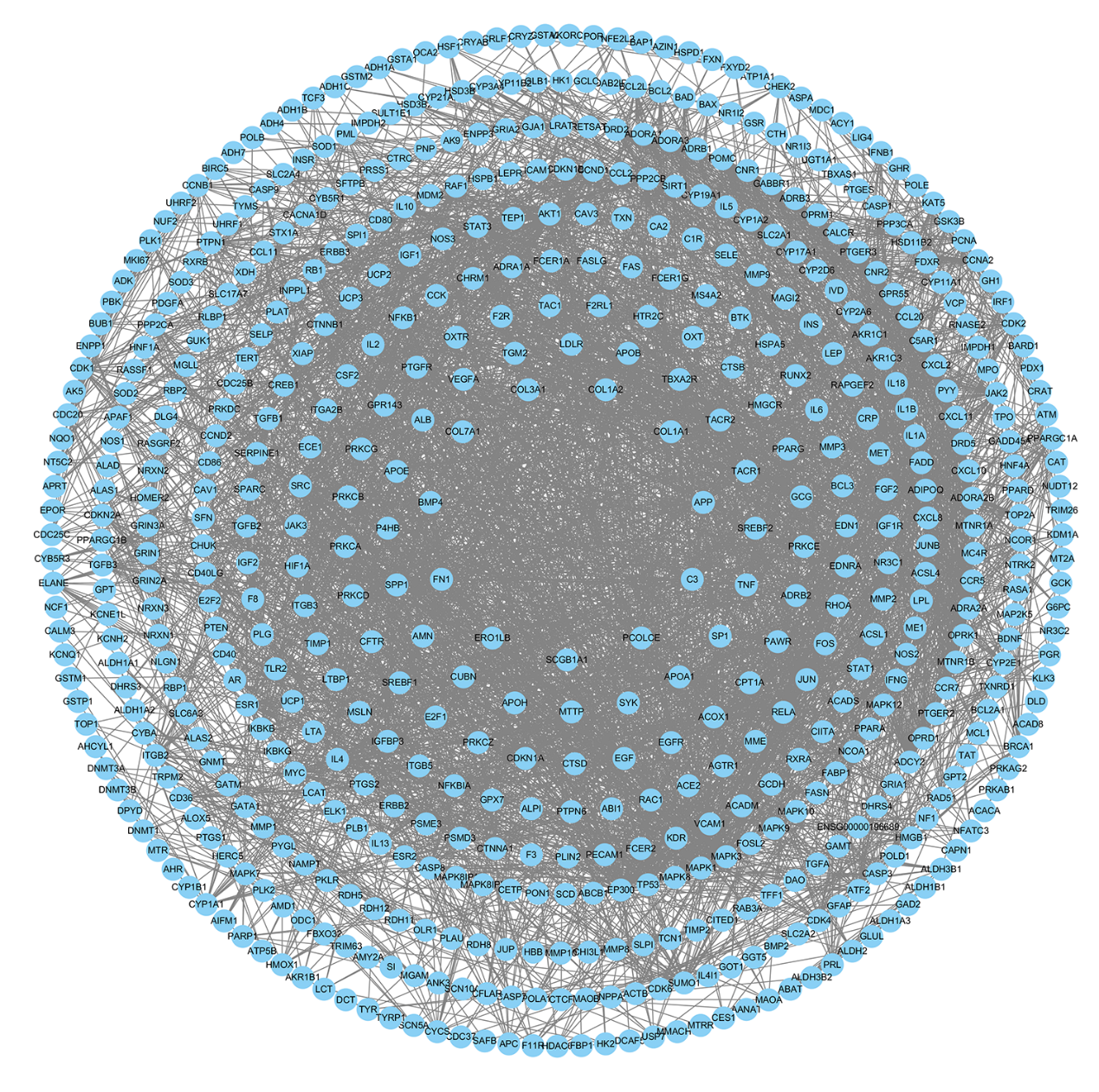


Figure S1: PPI network of Yixinyin targets. The figure is drawn by cytoscpe 3.7.0 software (https://cytoscape.org/).

Table S1: Clinical information of samples in GSE34198 data set

| Characteristic | | GSE34198(n=97) |
| --- | --- | --- |
| group | control | 48 |
|  | MI | 49 |
| sex | male | 65 |
|  | female | 32 |
| age | >60 | 39 |
|  | <=60 | 58 |
| diabetes status | no | 67 |
|  | yes | 30 |
| smoking status | no | 75 |
|  | yes | 22 |
| BMI | <24 | 6 |
|  | 24-28 | 27 |
|  | >28 | 56 |
| sbp [mmhg] | <=140 | 62 |
|  | >140 | 26 |
| dbp [mmhg] | <=90 | 80 |
|  | >90 | 8 |
| acei | no | 57 |
|  | yes | 40 |
| betablockers | no | 58 |
|  | yes | 39 |
| ca blockers | no | 70 |
|  | yes | 27 |
| statins | no | 62 |
|  | yes | 35 |
| fibrates | no | 93 |
|  | yes | 4 |
| other medication | no | 34 |
|  | yes | 63 |

Table S2: Enrichment analysis of differentially expressed genes

| down regulated genes in GSE66360 | | | | |
| --- | --- | --- | --- | --- |
| Term | Description | LogP | Log(q-value) | InTerm_InList |
| GO:0002274 | myeloid leukocyte activation | -59.09 | -54.73 | 81/661 |
| GO:0006954 | inflammatory response | -41.56 | -38.35 | 70/778 |
| GO:0009617 | response to bacterium | -33.25 | -30.07 | 60/728 |
| GO:0050900 | leukocyte migration | -26.98 | -23.87 | 46/511 |
| GO:0001817 | regulation of cytokine production | -26.04 | -23.00 | 54/782 |
| R-HSA-1280215 | Cytokine Signaling in Immune system | -25.21 | -22.19 | 51/715 |
| GO:0050865 | regulation of cell activation | -21.26 | -18.41 | 44/633 |
| hsa05140 | Leishmania infection | -20.24 | -17.45 | 19/73 |
| GO:0034341 | response to interferon-gamma | -18.34 | -15.60 | 25/197 |
| ko04380 | Osteoclast differentiation | -17.69 | -14.98 | 21/130 |
| ko04668 | TNF signaling pathway | -16.78 | -14.12 | 19/108 |
| GO:0006909 | phagocytosis | -15.08 | -12.50 | 29/382 |
| GO:0050778 | positive regulation of immune response | -14.39 | -11.85 | 39/761 |
| GO:0006959 | humoral immune response | -14.18 | -11.66 | 28/382 |
| GO:0030335 | positive regulation of cell migration | -14.12 | -11.60 | 33/550 |
| GO:0002521 | leukocyte differentiation | -13.87 | -11.37 | 32/526 |
| GO:2000379 | positive regulation of reactive oxygen species metabolic process | -13.47 | -11.00 | 16/101 |
| GO:0008285 | negative regulation of cell population proliferation | -13.10 | -10.68 | 37/753 |
| hsa05202 | Transcriptional misregulation in cancer | -12.60 | -10.24 | 20/204 |
| WP2882 | Nuclear Receptors Meta-Pathway | -12.38 | -10.04 | 24/322 |
| up regulated genes in GSE66360 | | | | |
| Term | Description | LogP | Log(q-value) | InTerm_InList |
| GO:0001706 | endoderm formation | -3.48 | 0.00 | 3/53 |
| R-HSA-380108 | Chemokine receptors bind chemokines | -3.35 | 0.00 | 3/59 |
| M54 | PID IL12 2PATHWAY | -3.28 | 0.00 | 3/62 |
| GO:0046631 | alpha-beta T cell activation | -3.26 | 0.00 | 4/149 |
| up regulated genes in GSE29111 | | | | |
| Term | Description | LogP | Log(q-value) | InTerm_InList |
| GO:0006911 | phagocytosis, engulfment | -7.26 | -3.17 | 8/126 |
| GO:0002576 | platelet degranulation | -3.72 | -0.72 | 5/128 |
| hsa04726 | Serotonergic synapse | -2.82 | -0.02 | 4/119 |
| GO:0001895 | retina homeostasis | -2.35 | 0.00 | 3/80 |
| GO:2000243 | positive regulation of reproductive process | -2.35 | 0.00 | 3/80 |
| hsa05150 | Staphylococcus aureus infection | -2.35 | 0.00 | 3/80 |
| hsa04970 | Salivary secretion | -2.16 | 0.00 | 3/94 |
| down regulated genes in GSE29111 | | | | |
| Term | Description | LogP | Log(q-value) | InTerm_InList |
| R-HSA-373076 | Class A/1 (Rhodopsin-like receptors) | -3.68 | 0.00 | 8/335 |
| GO:0070268 | cornification | -3.68 | 0.00 | 5/113 |
| GO:0002685 | regulation of leukocyte migration | -3.33 | 0.00 | 6/206 |
| WP4159 | GABA receptor Signaling | -3.29 | 0.00 | 3/33 |
| GO:0034314 | Arp2/3 complex-mediated actin nucleation | -3.07 | 0.00 | 3/39 |
| GO:0045666 | positive regulation of neuron differentiation | -2.97 | 0.00 | 4/94 |
| GO:0045926 | negative regulation of growth | -2.94 | 0.00 | 6/245 |
| GO:0120034 | positive regulation of plasma membrane bounded cell projection assembly | -2.78 | 0.00 | 4/106 |
| GO:0042493 | response to drug | -2.77 | 0.00 | 7/359 |
| GO:0006959 | humoral immune response | -2.62 | 0.00 | 7/382 |
| GO:0009952 | anterior/posterior pattern specification | -2.53 | 0.00 | 5/203 |
| M5885 | NABA MATRISOME ASSOCIATED | -2.49 | 0.00 | 10/751 |
| GO:0008285 | negative regulation of cell population proliferation | -2.48 | 0.00 | 10/753 |
| WP3998 | Prader-Willi and Angelman Syndrome | -2.41 | 0.00 | 3/66 |
| hsa04610 | Complement and coagulation cascades | -2.19 | 0.00 | 3/79 |
| GO:0048469 | cell maturation | -2.08 | 0.00 | 4/166 |

Table S3: Compound information of traditional Chinese medicine in Yixinyin

| compound Name | ID |
| --- | --- |
| Mandenol | MOL001494 |
| Linolenic acid ethyl ester | MOL007179 |
| senkyunone | MOL002151 |
| (Z)-3-(4-hydroxy-3-methoxy-phenyl)-N-[2-(4-hydroxyphenyl)ethyl]acrylamide | MOL000483 |
| 1-Monolinolein | MOL002464 |
| 3'-Hydroxymelanettin | MOL002958 |
| wallichilide | MOL002157 |
| Prostaglandin B1 | MOL007651 |
| neokadsuranic acid B | MOL009218 |
| Sitosteryl acetate | MOL001973 |
| Spinasterol | MOL004355 |
| Supraene | MOL001506 |
| isoduartin | MOL002985 |
| neokadsuranic acid A | MOL009217 |
| coumaroyltyramine | MOL000631 |
| 7-O-methylisomucronulatol | MOL000378 |
| 3-(2-hydroxy-3,4-dimethoxyphenyl)-2H-chromen-7-ol | MOL002997 |
| Schottenol | MOL006756 |
| Danshenol B | MOL007081 |
| (2R)-3-(3,4-dihydroxyphenyl)-2-[(Z)-3-(3,4-dihydroxyphenyl)acryloyl]oxy-propionic acid | MOL007132 |
| Eriodyctiol (flavanone) | MOL002914 |
| Danshenol A | MOL007082 |
| neokadsuranic acid C | MOL009219 |
| schizandronic acid | MOL009229 |
| n-coumaroyltyramine | MOL000332 |
| isomucronulatol-7,2'-di-O-glucosiole | MOL000439 |
| (3S,8S,9S,10R,13R,14S,17R)-10,13-dimethyl-17-[(2R,5S)-5-propan-2-yloctan-2-yl]-2,3,4,7,8,9,11,12,14,15,16,17-dodecahydro-1H-cyclopenta[a]phenanthren-3-ol | MOL000033 |
| Wuweizisu C | MOL008992 |
| (3R)-3-(2-hydroxy-3,4-dimethoxyphenyl)chroman-7-ol | MOL000438 |
| (3R)-3-(2,3-dihydroxy-4-methoxyphenyl)chroman-7,8-diol | MOL002941 |
| macrostemonoside e_qt | MOL007640 |
| Bifendate | MOL000387 |
| (6S)-6-(hydroxymethyl)-1,6-dimethyl-8,9-dihydro-7H-naphtho[8,7-g]benzofuran-10,11-dione | MOL007155 |
| kadsulactone | MOL009211 |
| (3S)-7-hydroxy-3-(2,3,4-trimethoxyphenyl)chroman-4-one | MOL002962 |
| changnanic acid | MOL009200 |
| Gomisin R | MOL008978 |
| beta-sitosterol | MOL000358 |
| 10α-cucurbita-5,24-diene-3β-ol | MOL007165 |
| (2R)-7-hydroxy-5-methoxy-2-phenylchroman-4-one | MOL000228 |
| (-)-Vestitol | MOL002961 |
| Deoxyharringtonine | MOL005317 |
| 4-Hydroxyhomopterocarpin | MOL002989 |
| dihydrotanshinlactone | MOL007100 |
| Sativanone | MOL002999 |
| (3R)-3-(2,3-dihydroxy-4-methoxyphenyl)-7-hydroxychroman-4-one | MOL002940 |
| poriferast-5-en-3beta-ol | MOL001771 |
| 2-(4-hydroxy-3-methoxyphenyl)-5-(3-hydroxypropyl)-7-methoxy-3-benzofurancarboxaldehyde | MOL007050 |
| (6aR,11aR)-3,9,10-trimethoxy-6a,11a-dihydro-6H-benzofurano[3,2-c]chromen-4-ol | MOL002990 |
| przewaquinone f | MOL007071 |
| (3R)-5'-Methoxyvestitol | MOL002939 |
| Xenognosin B | MOL003003 |
| isoimperatorin | MOL001942 |
| Baicalin | MOL002776 |
| naringenin | MOL004328 |
| 4',5',7-trimethyl-3-methoxyflavone | MOL002963 |
| schisanlactone A | MOL009222 |
| prolithospermic acid | MOL007130 |
| neocryptotanshinone | MOL007125 |
| angusifolin B | MOL009235 |
| vitamin-e | MOL007180 |
| digallate | MOL000569 |
| Salvilenone | MOL007085 |
| deoxyneocryptotanshinone | MOL007098 |
| Vestitone | MOL003001 |
| odoricarpin | MOL002996 |
| hederagenin | MOL000296 |
| Isotanshinone II | MOL007111 |
| violanone | MOL003002 |
| 7-hydroxy-4'-methoxy-2',5'-dioxo-4-[(3R)-2',7-dihydroxy-4'-methoxyisoflavan-5'-yl]isoflavane | MOL002967 |
| Duartin | MOL002981 |
| sitosterol | MOL000359 |
| quercetin | MOL000098 |
| dihydrotanshinoneⅠ | MOL007101 |
| FA | MOL000433 |
| Gomisin G | MOL008974 |
| przewaquinone c | MOL007069 |
| 9,10-dimethoxypterocarpan-3-O-β-D-glucoside | MOL000379 |
| Methylenetanshinquinone | MOL007061 |
| 3α-hydroxytanshinoneⅡa | MOL007045 |
| Kadsulignan C | MOL009210 |
| sugiol | MOL002222 |
| formononetin | MOL000392 |
| kadsulignan B | MOL009213 |
| manool | MOL007115 |
| Dehydrotanshinone II A | MOL002651 |
| (3R)-7,2',3'-trihydroxy-4-methoxyisoflavan | MOL002950 |
| α-amyrin | MOL006824 |
| salviolone | MOL007145 |
| 1,7-Dihydroxy-3,9-dimethoxy pterocarpene | MOL000442 |
| (6aR,11aR)-3,9-dimethoxy-6a,11a-dihydro-6H-benzofurano[3,2-c]chromene-4,10-diol | MOL002991 |
| 1,2,5,6-tetrahydrotanshinone | MOL001601 |
| 5'-hydroxyiso-muronulatol-2',5'-di-O-glucoside | MOL000374 |
| Hesperetin | MOL002341 |
| tanshinone Ⅵ | MOL007156 |
| przewalskin a | MOL007063 |
| C09092 | MOL007107 |
| Przewaquinone E | MOL007152 |
| formyltanshinone | MOL007058 |
| Calycosin | MOL000417 |
| 3-beta-Hydroxymethyllenetanshiquinone | MOL007059 |
| dan-shexinkum d | MOL007093 |
| Myricanone | MOL002135 |
| 3'-Methoxydaidzein | MOL002959 |
| (E)-3-[2-(3,4-dihydroxyphenyl)-7-hydroxy-benzofuran-4-yl]acrylic acid | MOL007048 |
| PGA(sup 1) | MOL007650 |
| miltipolone | MOL007121 |
| miltionone Ⅱ | MOL007120 |
| sclareol | MOL007077 |
| tanshinaldehyde | MOL007079 |
| Przewaquinone B | MOL007068 |
| epidanshenspiroketallactone | MOL007105 |
| Stevein | MOL003000 |
| (6S)-6-hydroxy-1-methyl-6-methylol-8,9-dihydro-7H-naphtho[8,7-g]benzofuran-10,11-quinone | MOL007150 |
| neokadsuranin | MOL009220 |
| miltirone Ⅱ | MOL007123 |
| Gomisin-A | MOL008968 |
| NSC 122421 | MOL007149 |
| isocryptotanshi-none | MOL007108 |
| (6S,7R)-6,7-dihydroxy-1,6-dimethyl-8,9-dihydro-7H-naphtho[8,7-g]benzofuran-10,11-dione | MOL007070 |
| 1-methyl-8,9-dihydro-7H-naphtho[5,6-g]benzofuran-6,10,11-trione | MOL007127 |
| Angeloylgomisin O | MOL008956 |
| 7-oxo-dihydrokaro-unidiol | MOL007172 |
| Linarin | MOL001790 |
| isoflavanone | MOL000398 |
| 9-O-Methylcoumestrol | MOL002957 |
| Tanshindiol B | MOL007151 |
| 5-dehydrokarounidiol | MOL007171 |
| przewalskin b | MOL007064 |
| Perlolyrine | MOL002140 |
| neocryptotanshinone ii | MOL007124 |
| isorhamnetin | MOL000354 |
| Jaranol | MOL000239 |
| Interiotherin B | MOL009199 |
| 2-isopropyl-8-methylphenanthrene-3,4-dione | MOL007041 |
| salvilenone Ⅰ | MOL007143 |
| DFV | MOL001792 |
| microstegiol | MOL007118 |
| Medicarpin | MOL002565 |
| tanshinone iia | MOL007154 |
| Taraxerol | MOL006554 |
| (6aR,11aR)-9,10-dimethoxy-6a,11a-dihydro-6H-benzofurano[3,2-c]chromen-3-ol | MOL000380 |
| acacetin | MOL001689 |
| (Z)-3-[2-[(E)-2-(3,4-dihydroxyphenyl)vinyl]-3,4-dihydroxy-phenyl]acrylic acid | MOL007140 |
| miltionone Ⅰ | MOL007119 |
| Schizandrer B | MOL008957 |
| 3,9-di-O-methylnissolin | MOL000371 |
| Bowdichione | MOL002973 |
| karounidiol 3-o-benzoate | MOL007175 |
| 4-methylenemiltirone | MOL007049 |
| butin | MOL002975 |
| danshenspiroketallactone | MOL007094 |
| (3R)-4'-Methoxy-2',3,7-trihydroxyisoflavanone | MOL002938 |
| salvianolic acid j | MOL007142 |
| Miltirone | MOL007122 |
| (3R,4R)-3',7-dihydroxy-2',4'-dimethoxy-4-[(2S)-4',5,7-trihydroxyflavanone-6-yl]isoflavan | MOL002982 |
| 6-o-syringyl-8-o-acetyl shanzhiside methyl ester | MOL007051 |
| luteolin | MOL000006 |
| Diosmetin | MOL002881 |
| Hydroxygenkwanin | MOL005530 |
| schisanlactone E | MOL009224 |
| 5,6-dihydroxy-7-isopropyl-1,1-dimethyl-2,3-dihydrophenanthren-4-one | MOL007036 |
| salvianolic acid g | MOL007141 |
| Mairin | MOL000211 |
| Poriferasterol | MOL001659 |
| kaempferol | MOL000422 |
| Longikaurin A | MOL004624 |
| (2R)-5,7-dihydroxy-2-(4-hydroxyphenyl)chroman-4-one | MOL001040 |
| Daidzein-4,7-diglucoside | MOL003629 |

Table S4: Docking results of Yixinyin compounds

| target | compound Name | ID | Total  Score |
| --- | --- | --- | --- |
| ALDH2 | Supraene | MOL001506 | 12.112 |
| ALDH2 | 1-Monolinolein | MOL002464 | 10.530 |
| ALDH2 | Mandenol | MOL001494 | 9.974 |
| ALDH2 | Linolenic acid ethyl ester | MOL007179 | 9.919 |
| ALDH2 | Linarin | MOL001790 | 8.076 |
| ALDH2 | PGA(sup 1) | MOL007650 | 7.858 |
| ALDH2 | FA | MOL000433 | 7.834 |
| ALDH2 | senkyunone | MOL002151 | 7.780 |
| ALDH2 | n-coumaroyltyramine | MOL000332 | 7.683 |
| ALDH2 | 3'-Methoxydaidzein | MOL002959 | 7.413 |
| ALDH2 | Prostaglandin B1 | MOL007651 | 7.171 |
| ALDH2 | quercetin | MOL000098 | 6.864 |
| ALDH2 | Calycosin | MOL000417 | 6.820 |
| ALDH2 | isomucronulatol-7,2'-di-O-glucosiole | MOL000439 | 6.692 |
| ALDH2 | 5'-hydroxyiso-muronulatol-2',5'-di-O-glucoside | MOL000374 | 6.664 |
| ALDH2 | vitamin-e | MOL007180 | 6.495 |
| ALDH2 | (2R)-3-(3,4-dihydroxyphenyl)-2-[(Z)-3-(3,4-dihydroxyphenyl)acryloyl]oxy-propionic acid | MOL007132 | 6.452 |
| ALDH2 | (Z)-3-[2-[(E)-2-(3,4-dihydroxyphenyl)vinyl]-3,4-dihydroxy-phenyl]acrylic acid | MOL007140 | 6.395 |
| ALDH2 | 2-(4-hydroxy-3-methoxyphenyl)-5-(3-hydroxypropyl)-7-methoxy-3-benzofurancarboxaldehyde | MOL007050 | 6.265 |
| ALDH2 | (Z)-3-(4-hydroxy-3-methoxy-phenyl)-N-[2-(4-hydroxyphenyl)ethyl]acrylamide | MOL000483 | 6.069 |
| ALDH2 | salvianolic acid j | MOL007142 | 5.976 |
| ALDH2 | Hesperetin | MOL002341 | 5.965 |
| ALDH2 | coumaroyltyramine | MOL000631 | 5.925 |
| ALDH2 | (3R)-3-(2,3-dihydroxy-4-methoxyphenyl)-7-hydroxychroman-4-one | MOL002940 | 5.803 |
| ALDH2 | formononetin | MOL000392 | 5.785 |
| ALDH2 | Xenognosin B | MOL003003 | 5.592 |
| ALDH2 | 4',5',7-trimethyl-3-methoxyflavone | MOL002963 | 5.572 |
| ALDH2 | neokadsuranic acid B | MOL009218 | 5.503 |
| ALDH2 | isorhamnetin | MOL000354 | 5.487 |
| ALDH2 | 3,9-di-O-methylnissolin | MOL000371 | 5.469 |
| ALDH2 | Baicalin | MOL002776 | 5.456 |
| ALDH2 | Sitosteryl acetate | MOL001973 | 5.435 |
| ALDH2 | (E)-3-[2-(3,4-dihydroxyphenyl)-7-hydroxy-benzofuran-4-yl]acrylic acid | MOL007048 | 5.381 |
| ALDH2 | isoduartin | MOL002985 | 5.371 |
| ALDH2 | (6aR,11aR)-3,9,10-trimethoxy-6a,11a-dihydro-6H-benzofurano[3,2-c]chromen-4-ol | MOL002990 | 5.358 |
| ALDH2 | 9,10-dimethoxypterocarpan-3-O-β-D-glucoside | MOL000379 | 5.341 |
| ALDH2 | (3R)-5'-Methoxyvestitol | MOL002939 | 5.302 |
| ALDH2 | Deoxyharringtonine | MOL005317 | 5.216 |
| ALDH2 | 7-hydroxy-4'-methoxy-2',5'-dioxo-4-[(3R)-2',7-dihydroxy-4'-methoxyisoflavan-5'-yl]isoflavane | MOL002967 | 5.185 |
| ALDH2 | Schottenol | MOL006756 | 5.161 |
| ALDH2 | Duartin | MOL002981 | 5.142 |
| ALDH2 | (-)-Vestitol | MOL002961 | 5.057 |
| ALDH2 | 2-isopropyl-8-methylphenanthrene-3,4-dione | MOL007041 | 5.054 |
| ALDH2 | wallichilide | MOL002157 | 5.025 |
| ALDH2 | acacetin | MOL001689 | 4.997 |
| ALDH2 | 10α-cucurbita-5,24-diene-3β-ol | MOL007165 | 4.975 |
| ALDH2 | prolithospermic acid | MOL007130 | 4.874 |
| ALDH2 | Vestitone | MOL003001 | 4.872 |
| ALDH2 | (3S)-7-hydroxy-3-(2,3,4-trimethoxyphenyl)chroman-4-one | MOL002962 | 4.849 |
| ALDH2 | (6aR,11aR)-9,10-dimethoxy-6a,11a-dihydro-6H-benzofurano[3,2-c]chromen-3-ol | MOL000380 | 4.805 |
| ALDH2 | (6aR,11aR)-3,9-dimethoxy-6a,11a-dihydro-6H-benzofurano[3,2-c]chromene-4,10-diol | MOL002991 | 4.776 |
| ALDH2 | 4-Hydroxyhomopterocarpin | MOL002989 | 4.698 |
| ALDH2 | Salvilenone | MOL007085 | 4.649 |
| ALDH2 | 1,7-Dihydroxy-3,9-dimethoxy pterocarpene | MOL000442 | 4.636 |
| ALDH2 | (3R)-3-(2-hydroxy-3,4-dimethoxyphenyl)chroman-7-ol | MOL000438 | 4.599 |
| ALDH2 | 3'-Hydroxymelanettin | MOL002958 | 4.481 |
| ALDH2 | hederagenin | MOL000296 | 4.449 |
| ALDH2 | salvianolic acid g | MOL007141 | 4.430 |
| ALDH2 | beta-sitosterol | MOL000358 | 4.388 |
| ALDH2 | Isotanshinone II | MOL007111 | 4.357 |
| ALDH2 | naringenin | MOL004328 | 4.348 |
| ALDH2 | neokadsuranic acid A | MOL009217 | 4.312 |
| ALDH2 | violanone | MOL003002 | 4.311 |
| ALDH2 | butin | MOL002975 | 4.289 |
| ALDH2 | Daidzein-4,7-diglucoside | MOL003629 | 4.267 |
| ALDH2 | Eriodyctiol (flavanone) | MOL002914 | 4.243 |
| ALDH2 | schisanlactone A | MOL009222 | 4.237 |
| ALDH2 | luteolin | MOL000006 | 4.203 |
| ALDH2 | kadsulactone | MOL009211 | 4.150 |
| ALDH2 | Perlolyrine | MOL002140 | 4.120 |
| ALDH2 | Przewaquinone B | MOL007068 | 4.119 |
| ALDH2 | (6S,7R)-6,7-dihydroxy-1,6-dimethyl-8,9-dihydro-7H-naphtho[8,7-g]benzofuran-10,11-dione | MOL007070 | 4.106 |
| ALDH2 | Bowdichione | MOL002973 | 4.087 |
| ALDH2 | digallate | MOL000569 | 4.054 |
| ALDH2 | tanshinone Ⅵ | MOL007156 | 4.044 |
| ALDH2 | (3R)-3-(2,3-dihydroxy-4-methoxyphenyl)chroman-7,8-diol | MOL002941 | 4.036 |
| ALDH2 | Sativanone | MOL002999 | 4.024 |
| ALDH2 | tanshinone iia | MOL007154 | 3.991 |
| ALDH2 | Jaranol | MOL000239 | 3.982 |
| ALDH2 | 3-(2-hydroxy-3,4-dimethoxyphenyl)-2H-chromen-7-ol | MOL002997 | 3.974 |
| ALDH2 | Poriferasterol | MOL001659 | 3.952 |
| ALDH2 | odoricarpin | MOL002996 | 3.908 |
| ALDH2 | Medicarpin | MOL002565 | 3.886 |
| ALDH2 | Stevein | MOL003000 | 3.844 |
| ALDH2 | kaempferol | MOL000422 | 3.836 |
| ALDH2 | miltirone Ⅱ | MOL007123 | 3.794 |
| ALDH2 | poriferast-5-en-3beta-ol | MOL001771 | 3.776 |
| ALDH2 | isoimperatorin | MOL001942 | 3.772 |
| ALDH2 | 1,2,5,6-tetrahydrotanshinone | MOL001601 | 3.755 |
| ALDH2 | (6S)-6-hydroxy-1-methyl-6-methylol-8,9-dihydro-7H-naphtho[8,7-g]benzofuran-10,11-quinone | MOL007150 | 3.741 |
| ALDH2 | schizandronic acid | MOL009229 | 3.692 |
| ALDH2 | dihydrotanshinoneⅠ | MOL007101 | 3.666 |
| ALDH2 | Tanshindiol B | MOL007151 | 3.627 |
| ALDH2 | (6S)-6-(hydroxymethyl)-1,6-dimethyl-8,9-dihydro-7H-naphtho[8,7-g]benzofuran-10,11-dione | MOL007155 | 3.610 |
| ALDH2 | isocryptotanshi-none | MOL007108 | 3.609 |
| ALDH2 | Gomisin R | MOL008978 | 3.567 |
| ALDH2 | Danshenol B | MOL007081 | 3.430 |
| ALDH2 | macrostemonoside e_qt | MOL007640 | 3.425 |
| ALDH2 | 5,6-dihydroxy-7-isopropyl-1,1-dimethyl-2,3-dihydrophenanthren-4-one | MOL007036 | 3.337 |
| ALDH2 | 6-o-syringyl-8-o-acetyl shanzhiside methyl ester | MOL007051 | 3.309 |
| ALDH2 | Bifendate | MOL000387 | 3.274 |
| ALDH2 | sclareol | MOL007077 | 3.272 |
| ALDH2 | (2R)-7-hydroxy-5-methoxy-2-phenylchroman-4-one | MOL000228 | 3.258 |
| ALDH2 | Hydroxygenkwanin | MOL005530 | 3.256 |
| ALDH2 | 9-O-Methylcoumestrol | MOL002957 | 3.253 |
| ALDH2 | Myricanone | MOL002135 | 3.215 |
| ALDH2 | isoflavanone | MOL000398 | 3.169 |
| ALDH2 | sugiol | MOL002222 | 3.162 |
| ALDH2 | Spinasterol | MOL004355 | 3.138 |
| ALDH2 | Diosmetin | MOL002881 | 3.075 |
| ALDH2 | neocryptotanshinone ii | MOL007124 | 3.058 |
| ALDH2 | Interiotherin B | MOL009199 | 3.031 |
| ALDH2 | przewalskin b | MOL007064 | 3.023 |
| ALDH2 | DFV | MOL001792 | 3.011 |
| ALDH2 | miltipolone | MOL007121 | 2.991 |
| ALDH2 | Schizandrer B | MOL008957 | 2.990 |
| ALDH2 | 3-beta-Hydroxymethyllenetanshiquinone | MOL007059 | 2.928 |
| ALDH2 | 7-O-methylisomucronulatol | MOL000378 | 2.893 |
| ALDH2 | salviolone | MOL007145 | 2.874 |
| ALDH2 | 3α-hydroxytanshinoneⅡa | MOL007045 | 2.856 |
| ALDH2 | Gomisin-A | MOL008968 | 2.843 |
| ALDH2 | miltionone Ⅱ | MOL007120 | 2.767 |
| ALDH2 | tanshinaldehyde | MOL007079 | 2.726 |
| ALDH2 | Danshenol A | MOL007082 | 2.704 |
| ALDH2 | Przewaquinone E | MOL007152 | 2.690 |
| ALDH2 | Miltirone | MOL007122 | 2.682 |
| ALDH2 | Angeloylgomisin O | MOL008956 | 2.679 |
| ALDH2 | przewaquinone c | MOL007069 | 2.676 |
| ALDH2 | sitosterol | MOL000359 | 2.662 |
| ALDH2 | Gomisin G | MOL008974 | 2.659 |
| ALDH2 | α-amyrin | MOL006824 | 2.636 |
| ALDH2 | neokadsuranic acid C | MOL009219 | 2.602 |
| ALDH2 | dihydrotanshinlactone | MOL007100 | 2.600 |
| ALDH2 | formyltanshinone | MOL007058 | 2.587 |
| ALDH2 | 4-methylenemiltirone | MOL007049 | 2.554 |
| ALDH2 | C09092 | MOL007107 | 2.519 |
| ALDH2 | epidanshenspiroketallactone | MOL007105 | 2.514 |
| ALDH2 | (2R)-5,7-dihydroxy-2-(4-hydroxyphenyl)chroman-4-one | MOL001040 | 2.507 |
| ALDH2 | Methylenetanshinquinone | MOL007061 | 2.485 |
| ALDH2 | microstegiol | MOL007118 | 2.437 |
| ALDH2 | przewaquinone f | MOL007071 | 2.417 |
| ALDH2 | (3R)-7,2',3'-trihydroxy-4-methoxyisoflavan | MOL002950 | 2.394 |
| ALDH2 | deoxyneocryptotanshinone | MOL007098 | 2.368 |
| ALDH2 | (3R,4R)-3',7-dihydroxy-2',4'-dimethoxy-4-[(2S)-4',5,7-trihydroxyflavanone-6-yl]isoflavan | MOL002982 | 2.358 |
| ALDH2 | Kadsulignan C | MOL009210 | 2.315 |
| ALDH2 | Wuweizisu C | MOL008992 | 2.309 |
| ALDH2 | przewalskin a | MOL007063 | 2.212 |
| ALDH2 | salvilenone Ⅰ | MOL007143 | 2.208 |
| ALDH2 | dan-shexinkum d | MOL007093 | 2.154 |
| ALDH2 | kadsulignan B | MOL009213 | 2.077 |
| ALDH2 | Taraxerol | MOL006554 | 2.049 |
| ALDH2 | miltionone Ⅰ | MOL007119 | 2.009 |
| ALDH2 | Dehydrotanshinone II A | MOL002651 | 1.978 |
| ALDH2 | neokadsuranin | MOL009220 | 1.954 |
| ALDH2 | neocryptotanshinone | MOL007125 | 1.908 |
| ALDH2 | 1-methyl-8,9-dihydro-7H-naphtho[5,6-g]benzofuran-6,10,11-trione | MOL007127 | 1.844 |
| ALDH2 | danshenspiroketallactone | MOL007094 | 1.804 |
| ALDH2 | (3R)-4'-Methoxy-2',3,7-trihydroxyisoflavanone | MOL002938 | 1.752 |
| ALDH2 | Longikaurin A | MOL004624 | 1.718 |
| ALDH2 | NSC 122421 | MOL007149 | 1.549 |
| ALDH2 | angusifolin B | MOL009235 | 1.315 |
| ALDH2 | 7-oxo-dihydrokaro-unidiol | MOL007172 | 0.598 |
| ALDH2 | manool | MOL007115 | -0.135 |
| ALDH2 | (3S,8S,9S,10R,13R,14S,17R)-10,13-dimethyl-17-[(2R,5S)-5-propan-2-yloctan-2-yl]-2,3,4,7,8,9,11,12,14,15,16,17-dodecahydro-1H-cyclopenta[a]phenanthren-3-ol | MOL000033 | -2.025 |
| ALDH2 | changnanic acid | MOL009200 | -4.598 |
| ALDH2 | 5-dehydrokarounidiol | MOL007171 | -4.771 |
| ALDH2 | karounidiol 3-o-benzoate | MOL007175 | -7.430 |
| ALDH2 | Mairin | MOL000211 | -11.419 |
| ALDH2 | schisanlactone E | MOL009224 | -11.447 |
| C5AR1 | Prostaglandin B1 | MOL007651 | 8.176 |
| C5AR1 | 1-Monolinolein | MOL002464 | 7.698 |
| C5AR1 | PGA(sup 1) | MOL007650 | 7.626 |
| C5AR1 | Mandenol | MOL001494 | 7.482 |
| C5AR1 | Supraene | MOL001506 | 6.967 |
| C5AR1 | salvianolic acid j | MOL007142 | 6.656 |
| C5AR1 | senkyunone | MOL002151 | 6.536 |
| C5AR1 | 5'-hydroxyiso-muronulatol-2',5'-di-O-glucoside | MOL000374 | 6.464 |
| C5AR1 | Linolenic acid ethyl ester | MOL007179 | 6.275 |
| C5AR1 | isomucronulatol-7,2'-di-O-glucosiole | MOL000439 | 5.955 |
| C5AR1 | Sitosteryl acetate | MOL001973 | 5.953 |
| C5AR1 | (2R)-3-(3,4-dihydroxyphenyl)-2-[(Z)-3-(3,4-dihydroxyphenyl)acryloyl]oxy-propionic acid | MOL007132 | 5.846 |
| C5AR1 | isoflavanone | MOL000398 | 5.805 |
| C5AR1 | neokadsuranic acid C | MOL009219 | 5.592 |
| C5AR1 | Angeloylgomisin O | MOL008956 | 5.506 |
| C5AR1 | 9,10-dimethoxypterocarpan-3-O-β-D-glucoside | MOL000379 | 5.404 |
| C5AR1 | n-coumaroyltyramine | MOL000332 | 5.267 |
| C5AR1 | Danshenol B | MOL007081 | 5.094 |
| C5AR1 | wallichilide | MOL002157 | 5.069 |
| C5AR1 | beta-sitosterol | MOL000358 | 5.060 |
| C5AR1 | changnanic acid | MOL009200 | 5.060 |
| C5AR1 | neokadsuranic acid A | MOL009217 | 5.027 |
| C5AR1 | 2-(4-hydroxy-3-methoxyphenyl)-5-(3-hydroxypropyl)-7-methoxy-3-benzofurancarboxaldehyde | MOL007050 | 4.992 |
| C5AR1 | (3S,8S,9S,10R,13R,14S,17R)-10,13-dimethyl-17-[(2R,5S)-5-propan-2-yloctan-2-yl]-2,3,4,7,8,9,11,12,14,15,16,17-dodecahydro-1H-cyclopenta[a]phenanthren-3-ol | MOL000033 | 4.932 |
| C5AR1 | FA | MOL000433 | 4.892 |
| C5AR1 | 10α-cucurbita-5,24-diene-3β-ol | MOL007165 | 4.871 |
| C5AR1 | violanone | MOL003002 | 4.843 |
| C5AR1 | isorhamnetin | MOL000354 | 4.822 |
| C5AR1 | (Z)-3-(4-hydroxy-3-methoxy-phenyl)-N-[2-(4-hydroxyphenyl)ethyl]acrylamide | MOL000483 | 4.819 |
| C5AR1 | Poriferasterol | MOL001659 | 4.810 |
| C5AR1 | Kadsulignan C | MOL009210 | 4.765 |
| C5AR1 | 7-O-methylisomucronulatol | MOL000378 | 4.721 |
| C5AR1 | (3R)-3-(2,3-dihydroxy-4-methoxyphenyl)chroman-7,8-diol | MOL002941 | 4.721 |
| C5AR1 | angusifolin B | MOL009235 | 4.717 |
| C5AR1 | neokadsuranic acid B | MOL009218 | 4.639 |
| C5AR1 | Deoxyharringtonine | MOL005317 | 4.554 |
| C5AR1 | hederagenin | MOL000296 | 4.551 |
| C5AR1 | Daidzein-4,7-diglucoside | MOL003629 | 4.537 |
| C5AR1 | (6aR,11aR)-3,9-dimethoxy-6a,11a-dihydro-6H-benzofurano[3,2-c]chromene-4,10-diol | MOL002991 | 4.518 |
| C5AR1 | macrostemonoside e_qt | MOL007640 | 4.505 |
| C5AR1 | Bifendate | MOL000387 | 4.469 |
| C5AR1 | Schottenol | MOL006756 | 4.458 |
| C5AR1 | Interiotherin B | MOL009199 | 4.397 |
| C5AR1 | (3R)-5'-Methoxyvestitol | MOL002939 | 4.381 |
| C5AR1 | schisanlactone A | MOL009222 | 4.349 |
| C5AR1 | Vestitone | MOL003001 | 4.341 |
| C5AR1 | (3R)-3-(2-hydroxy-3,4-dimethoxyphenyl)chroman-7-ol | MOL000438 | 4.318 |
| C5AR1 | Duartin | MOL002981 | 4.302 |
| C5AR1 | luteolin | MOL000006 | 4.249 |
| C5AR1 | Hesperetin | MOL002341 | 4.234 |
| C5AR1 | 5-dehydrokarounidiol | MOL007171 | 4.195 |
| C5AR1 | 3'-Methoxydaidzein | MOL002959 | 4.193 |
| C5AR1 | Baicalin | MOL002776 | 4.190 |
| C5AR1 | (E)-3-[2-(3,4-dihydroxyphenyl)-7-hydroxy-benzofuran-4-yl]acrylic acid | MOL007048 | 4.187 |
| C5AR1 | Gomisin-A | MOL008968 | 4.182 |
| C5AR1 | vitamin-e | MOL007180 | 4.181 |
| C5AR1 | digallate | MOL000569 | 4.153 |
| C5AR1 | isoimperatorin | MOL001942 | 4.124 |
| C5AR1 | (Z)-3-[2-[(E)-2-(3,4-dihydroxyphenyl)vinyl]-3,4-dihydroxy-phenyl]acrylic acid | MOL007140 | 4.105 |
| C5AR1 | Diosmetin | MOL002881 | 4.096 |
| C5AR1 | formononetin | MOL000392 | 4.057 |
| C5AR1 | 3'-Hydroxymelanettin | MOL002958 | 4.046 |
| C5AR1 | 4',5',7-trimethyl-3-methoxyflavone | MOL002963 | 4.034 |
| C5AR1 | Spinasterol | MOL004355 | 3.988 |
| C5AR1 | Xenognosin B | MOL003003 | 3.986 |
| C5AR1 | schizandronic acid | MOL009229 | 3.984 |
| C5AR1 | kadsulignan B | MOL009213 | 3.972 |
| C5AR1 | Jaranol | MOL000239 | 3.971 |
| C5AR1 | tanshinone iia | MOL007154 | 3.934 |
| C5AR1 | salvilenone Ⅰ | MOL007143 | 3.934 |
| C5AR1 | poriferast-5-en-3beta-ol | MOL001771 | 3.932 |
| C5AR1 | (3R)-7,2',3'-trihydroxy-4-methoxyisoflavan | MOL002950 | 3.920 |
| C5AR1 | (6S)-6-hydroxy-1-methyl-6-methylol-8,9-dihydro-7H-naphtho[8,7-g]benzofuran-10,11-quinone | MOL007150 | 3.910 |
| C5AR1 | coumaroyltyramine | MOL000631 | 3.900 |
| C5AR1 | Danshenol A | MOL007082 | 3.887 |
| C5AR1 | NSC 122421 | MOL007149 | 3.870 |
| C5AR1 | Linarin | MOL001790 | 3.814 |
| C5AR1 | (3R)-3-(2,3-dihydroxy-4-methoxyphenyl)-7-hydroxychroman-4-one | MOL002940 | 3.807 |
| C5AR1 | (3R,4R)-3',7-dihydroxy-2',4'-dimethoxy-4-[(2S)-4',5,7-trihydroxyflavanone-6-yl]isoflavan | MOL002982 | 3.794 |
| C5AR1 | 3,9-di-O-methylnissolin | MOL000371 | 3.760 |
| C5AR1 | kadsulactone | MOL009211 | 3.754 |
| C5AR1 | prolithospermic acid | MOL007130 | 3.747 |
| C5AR1 | salviolone | MOL007145 | 3.671 |
| C5AR1 | Calycosin | MOL000417 | 3.665 |
| C5AR1 | microstegiol | MOL007118 | 3.649 |
| C5AR1 | (2R)-5,7-dihydroxy-2-(4-hydroxyphenyl)chroman-4-one | MOL001040 | 3.623 |
| C5AR1 | Perlolyrine | MOL002140 | 3.607 |
| C5AR1 | 2-isopropyl-8-methylphenanthrene-3,4-dione | MOL007041 | 3.603 |
| C5AR1 | przewalskin b | MOL007064 | 3.579 |
| C5AR1 | Gomisin G | MOL008974 | 3.561 |
| C5AR1 | sitosterol | MOL000359 | 3.561 |
| C5AR1 | 1,7-Dihydroxy-3,9-dimethoxy pterocarpene | MOL000442 | 3.550 |
| C5AR1 | miltipolone | MOL007121 | 3.539 |
| C5AR1 | Eriodyctiol (flavanone) | MOL002914 | 3.527 |
| C5AR1 | odoricarpin | MOL002996 | 3.526 |
| C5AR1 | Schizandrer B | MOL008957 | 3.478 |
| C5AR1 | C09092 | MOL007107 | 3.437 |
| C5AR1 | isoduartin | MOL002985 | 3.412 |
| C5AR1 | Miltirone | MOL007122 | 3.410 |
| C5AR1 | acacetin | MOL001689 | 3.410 |
| C5AR1 | tanshinone Ⅵ | MOL007156 | 3.406 |
| C5AR1 | miltionone Ⅰ | MOL007119 | 3.403 |
| C5AR1 | Gomisin R | MOL008978 | 3.403 |
| C5AR1 | 3-(2-hydroxy-3,4-dimethoxyphenyl)-2H-chromen-7-ol | MOL002997 | 3.395 |
| C5AR1 | przewaquinone c | MOL007069 | 3.382 |
| C5AR1 | Isotanshinone II | MOL007111 | 3.373 |
| C5AR1 | deoxyneocryptotanshinone | MOL007098 | 3.355 |
| C5AR1 | isocryptotanshi-none | MOL007108 | 3.320 |
| C5AR1 | Salvilenone | MOL007085 | 3.303 |
| C5AR1 | Przewaquinone B | MOL007068 | 3.300 |
| C5AR1 | quercetin | MOL000098 | 3.295 |
| C5AR1 | Stevein | MOL003000 | 3.255 |
| C5AR1 | (2R)-7-hydroxy-5-methoxy-2-phenylchroman-4-one | MOL000228 | 3.249 |
| C5AR1 | 7-hydroxy-4'-methoxy-2',5'-dioxo-4-[(3R)-2',7-dihydroxy-4'-methoxyisoflavan-5'-yl]isoflavane | MOL002967 | 3.232 |
| C5AR1 | 4-Hydroxyhomopterocarpin | MOL002989 | 3.229 |
| C5AR1 | Hydroxygenkwanin | MOL005530 | 3.221 |
| C5AR1 | karounidiol 3-o-benzoate | MOL007175 | 3.220 |
| C5AR1 | Myricanone | MOL002135 | 3.219 |
| C5AR1 | salvianolic acid g | MOL007141 | 3.211 |
| C5AR1 | 4-methylenemiltirone | MOL007049 | 3.210 |
| C5AR1 | manool | MOL007115 | 3.205 |
| C5AR1 | neocryptotanshinone | MOL007125 | 3.195 |
| C5AR1 | miltionone Ⅱ | MOL007120 | 3.188 |
| C5AR1 | Medicarpin | MOL002565 | 3.179 |
| C5AR1 | (6aR,11aR)-9,10-dimethoxy-6a,11a-dihydro-6H-benzofurano[3,2-c]chromen-3-ol | MOL000380 | 3.173 |
| C5AR1 | Sativanone | MOL002999 | 3.171 |
| C5AR1 | Wuweizisu C | MOL008992 | 3.170 |
| C5AR1 | (6S,7R)-6,7-dihydroxy-1,6-dimethyl-8,9-dihydro-7H-naphtho[8,7-g]benzofuran-10,11-dione | MOL007070 | 3.165 |
| C5AR1 | epidanshenspiroketallactone | MOL007105 | 3.149 |
| C5AR1 | 3α-hydroxytanshinoneⅡa | MOL007045 | 3.112 |
| C5AR1 | (3S)-7-hydroxy-3-(2,3,4-trimethoxyphenyl)chroman-4-one | MOL002962 | 3.104 |
| C5AR1 | 5,6-dihydroxy-7-isopropyl-1,1-dimethyl-2,3-dihydrophenanthren-4-one | MOL007036 | 3.103 |
| C5AR1 | 9-O-Methylcoumestrol | MOL002957 | 3.085 |
| C5AR1 | przewaquinone f | MOL007071 | 3.074 |
| C5AR1 | sugiol | MOL002222 | 3.064 |
| C5AR1 | 1,2,5,6-tetrahydrotanshinone | MOL001601 | 2.964 |
| C5AR1 | butin | MOL002975 | 2.961 |
| C5AR1 | sclareol | MOL007077 | 2.954 |
| C5AR1 | (6S)-6-(hydroxymethyl)-1,6-dimethyl-8,9-dihydro-7H-naphtho[8,7-g]benzofuran-10,11-dione | MOL007155 | 2.908 |
| C5AR1 | dihydrotanshinoneⅠ | MOL007101 | 2.877 |
| C5AR1 | naringenin | MOL004328 | 2.873 |
| C5AR1 | 1-methyl-8,9-dihydro-7H-naphtho[5,6-g]benzofuran-6,10,11-trione | MOL007127 | 2.843 |
| C5AR1 | dan-shexinkum d | MOL007093 | 2.842 |
| C5AR1 | schisanlactone E | MOL009224 | 2.827 |
| C5AR1 | neokadsuranin | MOL009220 | 2.825 |
| C5AR1 | (6aR,11aR)-3,9,10-trimethoxy-6a,11a-dihydro-6H-benzofurano[3,2-c]chromen-4-ol | MOL002990 | 2.791 |
| C5AR1 | przewalskin a | MOL007063 | 2.790 |
| C5AR1 | danshenspiroketallactone | MOL007094 | 2.782 |
| C5AR1 | Bowdichione | MOL002973 | 2.742 |
| C5AR1 | tanshinaldehyde | MOL007079 | 2.741 |
| C5AR1 | formyltanshinone | MOL007058 | 2.718 |
| C5AR1 | DFV | MOL001792 | 2.701 |
| C5AR1 | miltirone Ⅱ | MOL007123 | 2.689 |
| C5AR1 | kaempferol | MOL000422 | 2.613 |
| C5AR1 | Taraxerol | MOL006554 | 2.591 |
| C5AR1 | dihydrotanshinlactone | MOL007100 | 2.553 |
| C5AR1 | Mairin | MOL000211 | 2.530 |
| C5AR1 | α-amyrin | MOL006824 | 2.527 |
| C5AR1 | Tanshindiol B | MOL007151 | 2.473 |
| C5AR1 | Przewaquinone E | MOL007152 | 2.458 |
| C5AR1 | neocryptotanshinone ii | MOL007124 | 2.434 |
| C5AR1 | Dehydrotanshinone II A | MOL002651 | 2.418 |
| C5AR1 | 3-beta-Hydroxymethyllenetanshiquinone | MOL007059 | 2.340 |
| C5AR1 | Methylenetanshinquinone | MOL007061 | 2.156 |
| C5AR1 | (3R)-4'-Methoxy-2',3,7-trihydroxyisoflavanone | MOL002938 | 2.094 |
| C5AR1 | 6-o-syringyl-8-o-acetyl shanzhiside methyl ester | MOL007051 | 2.000 |
| C5AR1 | Longikaurin A | MOL004624 | 1.896 |
| C5AR1 | 7-oxo-dihydrokaro-unidiol | MOL007172 | 1.801 |
| C5AR1 | (-)-Vestitol | MOL002961 | 1.727 |
| FOS | isomucronulatol-7,2'-di-O-glucosiole | MOL000439 | 7.639 |
| FOS | 5'-hydroxyiso-muronulatol-2',5'-di-O-glucoside | MOL000374 | 7.314 |
| FOS | Deoxyharringtonine | MOL005317 | 6.946 |
| FOS | Mandenol | MOL001494 | 6.911 |
| FOS | Supraene | MOL001506 | 6.757 |
| FOS | Interiotherin B | MOL009199 | 6.665 |
| FOS | Angeloylgomisin O | MOL008956 | 6.655 |
| FOS | (2R)-3-(3,4-dihydroxyphenyl)-2-[(Z)-3-(3,4-dihydroxyphenyl)acryloyl]oxy-propionic acid | MOL007132 | 6.423 |
| FOS | kadsulignan B | MOL009213 | 6.382 |
| FOS | Linolenic acid ethyl ester | MOL007179 | 6.320 |
| FOS | schizandronic acid | MOL009229 | 6.308 |
| FOS | Baicalin | MOL002776 | 6.277 |
| FOS | FA | MOL000433 | 6.197 |
| FOS | changnanic acid | MOL009200 | 6.137 |
| FOS | Prostaglandin B1 | MOL007651 | 6.051 |
| FOS | wallichilide | MOL002157 | 5.966 |
| FOS | schisanlactone E | MOL009224 | 5.899 |
| FOS | Stevein | MOL003000 | 5.888 |
| FOS | senkyunone | MOL002151 | 5.824 |
| FOS | neokadsuranic acid B | MOL009218 | 5.798 |
| FOS | PGA(sup 1) | MOL007650 | 5.781 |
| FOS | neokadsuranic acid A | MOL009217 | 5.756 |
| FOS | (6S,7R)-6,7-dihydroxy-1,6-dimethyl-8,9-dihydro-7H-naphtho[8,7-g]benzofuran-10,11-dione | MOL007070 | 5.625 |
| FOS | 1-Monolinolein | MOL002464 | 5.593 |
| FOS | Schizandrer B | MOL008957 | 5.559 |
| FOS | przewaquinone c | MOL007069 | 5.534 |
| FOS | Bifendate | MOL000387 | 5.469 |
| FOS | Linarin | MOL001790 | 5.446 |
| FOS | 9,10-dimethoxypterocarpan-3-O-β-D-glucoside | MOL000379 | 5.415 |
| FOS | prolithospermic acid | MOL007130 | 5.381 |
| FOS | (3R)-7,2',3'-trihydroxy-4-methoxyisoflavan | MOL002950 | 5.347 |
| FOS | Danshenol A | MOL007082 | 5.304 |
| FOS | (E)-3-[2-(3,4-dihydroxyphenyl)-7-hydroxy-benzofuran-4-yl]acrylic acid | MOL007048 | 5.265 |
| FOS | Wuweizisu C | MOL008992 | 5.235 |
| FOS | (6aR,11aR)-9,10-dimethoxy-6a,11a-dihydro-6H-benzofurano[3,2-c]chromen-3-ol | MOL000380 | 5.230 |
| FOS | neokadsuranin | MOL009220 | 5.198 |
| FOS | Jaranol | MOL000239 | 5.176 |
| FOS | schisanlactone A | MOL009222 | 5.134 |
| FOS | sitosterol | MOL000359 | 5.093 |
| FOS | salvianolic acid j | MOL007142 | 4.985 |
| FOS | 4-methylenemiltirone | MOL007049 | 4.967 |
| FOS | poriferast-5-en-3beta-ol | MOL001771 | 4.940 |
| FOS | 7-oxo-dihydrokaro-unidiol | MOL007172 | 4.935 |
| FOS | vitamin-e | MOL007180 | 4.921 |
| FOS | (6S)-6-(hydroxymethyl)-1,6-dimethyl-8,9-dihydro-7H-naphtho[8,7-g]benzofuran-10,11-dione | MOL007155 | 4.904 |
| FOS | isorhamnetin | MOL000354 | 4.900 |
| FOS | 4',5',7-trimethyl-3-methoxyflavone | MOL002963 | 4.879 |
| FOS | 2-(4-hydroxy-3-methoxyphenyl)-5-(3-hydroxypropyl)-7-methoxy-3-benzofurancarboxaldehyde | MOL007050 | 4.802 |
| FOS | (6S)-6-hydroxy-1-methyl-6-methylol-8,9-dihydro-7H-naphtho[8,7-g]benzofuran-10,11-quinone | MOL007150 | 4.787 |
| FOS | miltipolone | MOL007121 | 4.776 |
| FOS | przewalskin a | MOL007063 | 4.773 |
| FOS | beta-sitosterol | MOL000358 | 4.768 |
| FOS | tanshinaldehyde | MOL007079 | 4.768 |
| FOS | Gomisin-A | MOL008968 | 4.742 |
| FOS | angusifolin B | MOL009235 | 4.733 |
| FOS | tanshinone Ⅵ | MOL007156 | 4.707 |
| FOS | 5-dehydrokarounidiol | MOL007171 | 4.699 |
| FOS | (3R)-5'-Methoxyvestitol | MOL002939 | 4.667 |
| FOS | naringenin | MOL004328 | 4.639 |
| FOS | Sativanone | MOL002999 | 4.628 |
| FOS | deoxyneocryptotanshinone | MOL007098 | 4.613 |
| FOS | kadsulactone | MOL009211 | 4.597 |
| FOS | manool | MOL007115 | 4.590 |
| FOS | 1-methyl-8,9-dihydro-7H-naphtho[5,6-g]benzofuran-6,10,11-trione | MOL007127 | 4.560 |
| FOS | (3S)-7-hydroxy-3-(2,3,4-trimethoxyphenyl)chroman-4-one | MOL002962 | 4.515 |
| FOS | (6aR,11aR)-3,9-dimethoxy-6a,11a-dihydro-6H-benzofurano[3,2-c]chromene-4,10-diol | MOL002991 | 4.497 |
| FOS | przewaquinone f | MOL007071 | 4.488 |
| FOS | 3'-Methoxydaidzein | MOL002959 | 4.478 |
| FOS | Vestitone | MOL003001 | 4.467 |
| FOS | digallate | MOL000569 | 4.458 |
| FOS | Sitosteryl acetate | MOL001973 | 4.451 |
| FOS | Poriferasterol | MOL001659 | 4.449 |
| FOS | Danshenol B | MOL007081 | 4.428 |
| FOS | Schottenol | MOL006756 | 4.421 |
| FOS | Hesperetin | MOL002341 | 4.418 |
| FOS | (3S,8S,9S,10R,13R,14S,17R)-10,13-dimethyl-17-[(2R,5S)-5-propan-2-yloctan-2-yl]-2,3,4,7,8,9,11,12,14,15,16,17-dodecahydro-1H-cyclopenta[a]phenanthren-3-ol | MOL000033 | 4.414 |
| FOS | (2R)-7-hydroxy-5-methoxy-2-phenylchroman-4-one | MOL000228 | 4.329 |
| FOS | miltionone Ⅱ | MOL007120 | 4.325 |
| FOS | (3R)-3-(2,3-dihydroxy-4-methoxyphenyl)chroman-7,8-diol | MOL002941 | 4.306 |
| FOS | 6-o-syringyl-8-o-acetyl shanzhiside methyl ester | MOL007051 | 4.287 |
| FOS | salvianolic acid g | MOL007141 | 4.254 |
| FOS | n-coumaroyltyramine | MOL000332 | 4.254 |
| FOS | isoimperatorin | MOL001942 | 4.247 |
| FOS | (3R)-3-(2-hydroxy-3,4-dimethoxyphenyl)chroman-7-ol | MOL000438 | 4.216 |
| FOS | Myricanone | MOL002135 | 4.209 |
| FOS | hederagenin | MOL000296 | 4.197 |
| FOS | Tanshindiol B | MOL007151 | 4.195 |
| FOS | coumaroyltyramine | MOL000631 | 4.176 |
| FOS | isoflavanone | MOL000398 | 4.174 |
| FOS | (Z)-3-(4-hydroxy-3-methoxy-phenyl)-N-[2-(4-hydroxyphenyl)ethyl]acrylamide | MOL000483 | 4.172 |
| FOS | 3,9-di-O-methylnissolin | MOL000371 | 4.157 |
| FOS | (Z)-3-[2-[(E)-2-(3,4-dihydroxyphenyl)vinyl]-3,4-dihydroxy-phenyl]acrylic acid | MOL007140 | 4.135 |
| FOS | isoduartin | MOL002985 | 4.134 |
| FOS | sclareol | MOL007077 | 4.124 |
| FOS | 1,2,5,6-tetrahydrotanshinone | MOL001601 | 4.119 |
| FOS | odoricarpin | MOL002996 | 4.111 |
| FOS | Salvilenone | MOL007085 | 4.086 |
| FOS | Taraxerol | MOL006554 | 4.065 |
| FOS | Medicarpin | MOL002565 | 4.037 |
| FOS | przewalskin b | MOL007064 | 4.014 |
| FOS | tanshinone iia | MOL007154 | 3.999 |
| FOS | Spinasterol | MOL004355 | 3.997 |
| FOS | 3'-Hydroxymelanettin | MOL002958 | 3.986 |
| FOS | neocryptotanshinone | MOL007125 | 3.983 |
| FOS | dan-shexinkum d | MOL007093 | 3.982 |
| FOS | 7-O-methylisomucronulatol | MOL000378 | 3.978 |
| FOS | Isotanshinone II | MOL007111 | 3.956 |
| FOS | Bowdichione | MOL002973 | 3.925 |
| FOS | Calycosin | MOL000417 | 3.902 |
| FOS | (3R)-3-(2,3-dihydroxy-4-methoxyphenyl)-7-hydroxychroman-4-one | MOL002940 | 3.901 |
| FOS | epidanshenspiroketallactone | MOL007105 | 3.850 |
| FOS | Duartin | MOL002981 | 3.846 |
| FOS | Gomisin G | MOL008974 | 3.822 |
| FOS | dihydrotanshinoneⅠ | MOL007101 | 3.820 |
| FOS | 3-beta-Hydroxymethyllenetanshiquinone | MOL007059 | 3.809 |
| FOS | 3α-hydroxytanshinoneⅡa | MOL007045 | 3.796 |
| FOS | Xenognosin B | MOL003003 | 3.781 |
| FOS | (-)-Vestitol | MOL002961 | 3.755 |
| FOS | dihydrotanshinlactone | MOL007100 | 3.739 |
| FOS | (6aR,11aR)-3,9,10-trimethoxy-6a,11a-dihydro-6H-benzofurano[3,2-c]chromen-4-ol | MOL002990 | 3.738 |
| FOS | formyltanshinone | MOL007058 | 3.732 |
| FOS | danshenspiroketallactone | MOL007094 | 3.715 |
| FOS | (3R)-4'-Methoxy-2',3,7-trihydroxyisoflavanone | MOL002938 | 3.710 |
| FOS | violanone | MOL003002 | 3.707 |
| FOS | neokadsuranic acid C | MOL009219 | 3.694 |
| FOS | Eriodyctiol (flavanone) | MOL002914 | 3.692 |
| FOS | Miltirone | MOL007122 | 3.688 |
| FOS | isocryptotanshi-none | MOL007108 | 3.688 |
| FOS | Perlolyrine | MOL002140 | 3.644 |
| FOS | Dehydrotanshinone II A | MOL002651 | 3.639 |
| FOS | 3-(2-hydroxy-3,4-dimethoxyphenyl)-2H-chromen-7-ol | MOL002997 | 3.638 |
| FOS | Przewaquinone B | MOL007068 | 3.630 |
| FOS | miltirone Ⅱ | MOL007123 | 3.531 |
| FOS | Longikaurin A | MOL004624 | 3.507 |
| FOS | 9-O-Methylcoumestrol | MOL002957 | 3.478 |
| FOS | salvilenone Ⅰ | MOL007143 | 3.449 |
| FOS | 7-hydroxy-4'-methoxy-2',5'-dioxo-4-[(3R)-2',7-dihydroxy-4'-methoxyisoflavan-5'-yl]isoflavane | MOL002967 | 3.435 |
| FOS | NSC 122421 | MOL007149 | 3.431 |
| FOS | karounidiol 3-o-benzoate | MOL007175 | 3.400 |
| FOS | 4-Hydroxyhomopterocarpin | MOL002989 | 3.389 |
| FOS | neocryptotanshinone ii | MOL007124 | 3.339 |
| FOS | 1,7-Dihydroxy-3,9-dimethoxy pterocarpene | MOL000442 | 3.303 |
| FOS | 2-isopropyl-8-methylphenanthrene-3,4-dione | MOL007041 | 3.298 |
| FOS | kaempferol | MOL000422 | 3.229 |
| FOS | quercetin | MOL000098 | 3.226 |
| FOS | miltionone Ⅰ | MOL007119 | 3.222 |
| FOS | 5,6-dihydroxy-7-isopropyl-1,1-dimethyl-2,3-dihydrophenanthren-4-one | MOL007036 | 3.206 |
| FOS | Kadsulignan C | MOL009210 | 3.202 |
| FOS | luteolin | MOL000006 | 3.185 |
| FOS | butin | MOL002975 | 3.182 |
| FOS | Methylenetanshinquinone | MOL007061 | 3.173 |
| FOS | Diosmetin | MOL002881 | 3.134 |
| FOS | acacetin | MOL001689 | 3.029 |
| FOS | 10α-cucurbita-5,24-diene-3β-ol | MOL007165 | 3.021 |
| FOS | macrostemonoside e_qt | MOL007640 | 2.997 |
| FOS | α-amyrin | MOL006824 | 2.977 |
| FOS | (3R,4R)-3',7-dihydroxy-2',4'-dimethoxy-4-[(2S)-4',5,7-trihydroxyflavanone-6-yl]isoflavan | MOL002982 | 2.962 |
| FOS | salviolone | MOL007145 | 2.948 |
| FOS | Mairin | MOL000211 | 2.943 |
| FOS | DFV | MOL001792 | 2.918 |
| FOS | Hydroxygenkwanin | MOL005530 | 2.853 |
| FOS | Przewaquinone E | MOL007152 | 2.807 |
| FOS | microstegiol | MOL007118 | 2.803 |
| FOS | formononetin | MOL000392 | 2.707 |
| FOS | Daidzein-4,7-diglucoside | MOL003629 | 2.687 |
| FOS | Gomisin R | MOL008978 | 2.673 |
| FOS | (2R)-5,7-dihydroxy-2-(4-hydroxyphenyl)chroman-4-one | MOL001040 | 2.617 |
| FOS | C09092 | MOL007107 | 2.500 |
| FOS | sugiol | MOL002222 | 2.259 |
| IL1B | angusifolin B | MOL009235 | 6.475 |
| IL1B | 2-(4-hydroxy-3-methoxyphenyl)-5-(3-hydroxypropyl)-7-methoxy-3-benzofurancarboxaldehyde | MOL007050 | 6.217 |
| IL1B | Deoxyharringtonine | MOL005317 | 6.112 |
| IL1B | isorhamnetin | MOL000354 | 5.989 |
| IL1B | Linolenic acid ethyl ester | MOL007179 | 5.987 |
| IL1B | vitamin-e | MOL007180 | 5.676 |
| IL1B | Bifendate | MOL000387 | 5.608 |
| IL1B | Prostaglandin B1 | MOL007651 | 5.459 |
| IL1B | Mandenol | MOL001494 | 5.420 |
| IL1B | isomucronulatol-7,2'-di-O-glucosiole | MOL000439 | 5.413 |
| IL1B | quercetin | MOL000098 | 5.386 |
| IL1B | 1-Monolinolein | MOL002464 | 5.327 |
| IL1B | FA | MOL000433 | 5.222 |
| IL1B | 9,10-dimethoxypterocarpan-3-O-β-D-glucoside | MOL000379 | 5.196 |
| IL1B | (2R)-3-(3,4-dihydroxyphenyl)-2-[(Z)-3-(3,4-dihydroxyphenyl)acryloyl]oxy-propionic acid | MOL007132 | 5.154 |
| IL1B | (3R)-3-(2,3-dihydroxy-4-methoxyphenyl)chroman-7,8-diol | MOL002941 | 5.153 |
| IL1B | (2R)-5,7-dihydroxy-2-(4-hydroxyphenyl)chroman-4-one | MOL001040 | 5.049 |
| IL1B | (Z)-3-(4-hydroxy-3-methoxy-phenyl)-N-[2-(4-hydroxyphenyl)ethyl]acrylamide | MOL000483 | 5.027 |
| IL1B | 3-(2-hydroxy-3,4-dimethoxyphenyl)-2H-chromen-7-ol | MOL002997 | 4.984 |
| IL1B | kadsulactone | MOL009211 | 4.942 |
| IL1B | manool | MOL007115 | 4.929 |
| IL1B | Supraene | MOL001506 | 4.883 |
| IL1B | sclareol | MOL007077 | 4.881 |
| IL1B | changnanic acid | MOL009200 | 4.848 |
| IL1B | wallichilide | MOL002157 | 4.817 |
| IL1B | 7-O-methylisomucronulatol | MOL000378 | 4.813 |
| IL1B | kadsulignan B | MOL009213 | 4.776 |
| IL1B | Vestitone | MOL003001 | 4.705 |
| IL1B | Duartin | MOL002981 | 4.662 |
| IL1B | Sativanone | MOL002999 | 4.654 |
| IL1B | (3R)-3-(2-hydroxy-3,4-dimethoxyphenyl)chroman-7-ol | MOL000438 | 4.641 |
| IL1B | neokadsuranic acid C | MOL009219 | 4.612 |
| IL1B | PGA(sup 1) | MOL007650 | 4.607 |
| IL1B | neokadsuranic acid A | MOL009217 | 4.600 |
| IL1B | beta-sitosterol | MOL000358 | 4.598 |
| IL1B | (6aR,11aR)-3,9,10-trimethoxy-6a,11a-dihydro-6H-benzofurano[3,2-c]chromen-4-ol | MOL002990 | 4.587 |
| IL1B | 6-o-syringyl-8-o-acetyl shanzhiside methyl ester | MOL007051 | 4.536 |
| IL1B | (E)-3-[2-(3,4-dihydroxyphenyl)-7-hydroxy-benzofuran-4-yl]acrylic acid | MOL007048 | 4.519 |
| IL1B | Hesperetin | MOL002341 | 4.511 |
| IL1B | (3R)-4'-Methoxy-2',3,7-trihydroxyisoflavanone | MOL002938 | 4.485 |
| IL1B | (3R)-7,2',3'-trihydroxy-4-methoxyisoflavan | MOL002950 | 4.444 |
| IL1B | Myricanone | MOL002135 | 4.424 |
| IL1B | (3S)-7-hydroxy-3-(2,3,4-trimethoxyphenyl)chroman-4-one | MOL002962 | 4.415 |
| IL1B | Linarin | MOL001790 | 4.414 |
| IL1B | digallate | MOL000569 | 4.364 |
| IL1B | Kadsulignan C | MOL009210 | 4.351 |
| IL1B | schizandronic acid | MOL009229 | 4.334 |
| IL1B | 3,9-di-O-methylnissolin | MOL000371 | 4.312 |
| IL1B | 3'-Hydroxymelanettin | MOL002958 | 4.266 |
| IL1B | Angeloylgomisin O | MOL008956 | 4.262 |
| IL1B | Poriferasterol | MOL001659 | 4.259 |
| IL1B | Jaranol | MOL000239 | 4.208 |
| IL1B | Danshenol B | MOL007081 | 4.192 |
| IL1B | neokadsuranic acid B | MOL009218 | 4.184 |
| IL1B | salvilenone Ⅰ | MOL007143 | 4.174 |
| IL1B | (6S,7R)-6,7-dihydroxy-1,6-dimethyl-8,9-dihydro-7H-naphtho[8,7-g]benzofuran-10,11-dione | MOL007070 | 4.159 |
| IL1B | Danshenol A | MOL007082 | 4.156 |
| IL1B | Schottenol | MOL006756 | 4.137 |
| IL1B | odoricarpin | MOL002996 | 4.128 |
| IL1B | przewalskin a | MOL007063 | 4.107 |
| IL1B | C09092 | MOL007107 | 4.105 |
| IL1B | coumaroyltyramine | MOL000631 | 4.088 |
| IL1B | isoflavanone | MOL000398 | 4.059 |
| IL1B | hederagenin | MOL000296 | 4.059 |
| IL1B | 3'-Methoxydaidzein | MOL002959 | 4.044 |
| IL1B | (6aR,11aR)-3,9-dimethoxy-6a,11a-dihydro-6H-benzofurano[3,2-c]chromene-4,10-diol | MOL002991 | 4.003 |
| IL1B | (-)-Vestitol | MOL002961 | 4.001 |
| IL1B | (3R)-5'-Methoxyvestitol | MOL002939 | 3.978 |
| IL1B | sitosterol | MOL000359 | 3.940 |
| IL1B | Eriodyctiol (flavanone) | MOL002914 | 3.926 |
| IL1B | microstegiol | MOL007118 | 3.908 |
| IL1B | Perlolyrine | MOL002140 | 3.902 |
| IL1B | (3R)-3-(2,3-dihydroxy-4-methoxyphenyl)-7-hydroxychroman-4-one | MOL002940 | 3.900 |
| IL1B | (6aR,11aR)-9,10-dimethoxy-6a,11a-dihydro-6H-benzofurano[3,2-c]chromen-3-ol | MOL000380 | 3.890 |
| IL1B | Sitosteryl acetate | MOL001973 | 3.888 |
| IL1B | (3R,4R)-3',7-dihydroxy-2',4'-dimethoxy-4-[(2S)-4',5,7-trihydroxyflavanone-6-yl]isoflavan | MOL002982 | 3.884 |
| IL1B | macrostemonoside e_qt | MOL007640 | 3.881 |
| IL1B | salviolone | MOL007145 | 3.847 |
| IL1B | 4',5',7-trimethyl-3-methoxyflavone | MOL002963 | 3.843 |
| IL1B | neocryptotanshinone | MOL007125 | 3.823 |
| IL1B | isoimperatorin | MOL001942 | 3.820 |
| IL1B | violanone | MOL003002 | 3.803 |
| IL1B | n-coumaroyltyramine | MOL000332 | 3.799 |
| IL1B | neocryptotanshinone ii | MOL007124 | 3.760 |
| IL1B | Baicalin | MOL002776 | 3.750 |
| IL1B | (Z)-3-[2-[(E)-2-(3,4-dihydroxyphenyl)vinyl]-3,4-dihydroxy-phenyl]acrylic acid | MOL007140 | 3.749 |
| IL1B | Interiotherin B | MOL009199 | 3.727 |
| IL1B | 9-O-Methylcoumestrol | MOL002957 | 3.720 |
| IL1B | karounidiol 3-o-benzoate | MOL007175 | 3.709 |
| IL1B | 10α-cucurbita-5,24-diene-3β-ol | MOL007165 | 3.708 |
| IL1B | 2-isopropyl-8-methylphenanthrene-3,4-dione | MOL007041 | 3.670 |
| IL1B | 4-Hydroxyhomopterocarpin | MOL002989 | 3.642 |
| IL1B | (3S,8S,9S,10R,13R,14S,17R)-10,13-dimethyl-17-[(2R,5S)-5-propan-2-yloctan-2-yl]-2,3,4,7,8,9,11,12,14,15,16,17-dodecahydro-1H-cyclopenta[a]phenanthren-3-ol | MOL000033 | 3.618 |
| IL1B | Stevein | MOL003000 | 3.604 |
| IL1B | Miltirone | MOL007122 | 3.596 |
| IL1B | 5,6-dihydroxy-7-isopropyl-1,1-dimethyl-2,3-dihydrophenanthren-4-one | MOL007036 | 3.580 |
| IL1B | Schizandrer B | MOL008957 | 3.567 |
| IL1B | 1,7-Dihydroxy-3,9-dimethoxy pterocarpene | MOL000442 | 3.563 |
| IL1B | isoduartin | MOL002985 | 3.535 |
| IL1B | dan-shexinkum d | MOL007093 | 3.525 |
| IL1B | prolithospermic acid | MOL007130 | 3.501 |
| IL1B | Calycosin | MOL000417 | 3.473 |
| IL1B | kaempferol | MOL000422 | 3.448 |
| IL1B | 5'-hydroxyiso-muronulatol-2',5'-di-O-glucoside | MOL000374 | 3.430 |
| IL1B | (6S)-6-(hydroxymethyl)-1,6-dimethyl-8,9-dihydro-7H-naphtho[8,7-g]benzofuran-10,11-dione | MOL007155 | 3.417 |
| IL1B | Spinasterol | MOL004355 | 3.403 |
| IL1B | Tanshindiol B | MOL007151 | 3.379 |
| IL1B | schisanlactone A | MOL009222 | 3.351 |
| IL1B | Daidzein-4,7-diglucoside | MOL003629 | 3.348 |
| IL1B | sugiol | MOL002222 | 3.347 |
| IL1B | salvianolic acid j | MOL007142 | 3.345 |
| IL1B | przewalskin b | MOL007064 | 3.343 |
| IL1B | Salvilenone | MOL007085 | 3.314 |
| IL1B | deoxyneocryptotanshinone | MOL007098 | 3.304 |
| IL1B | miltipolone | MOL007121 | 3.302 |
| IL1B | 4-methylenemiltirone | MOL007049 | 3.302 |
| IL1B | Xenognosin B | MOL003003 | 3.291 |
| IL1B | Wuweizisu C | MOL008992 | 3.271 |
| IL1B | 7-hydroxy-4'-methoxy-2',5'-dioxo-4-[(3R)-2',7-dihydroxy-4'-methoxyisoflavan-5'-yl]isoflavane | MOL002967 | 3.269 |
| IL1B | Gomisin-A | MOL008968 | 3.261 |
| IL1B | Gomisin R | MOL008978 | 3.242 |
| IL1B | 7-oxo-dihydrokaro-unidiol | MOL007172 | 3.239 |
| IL1B | Methylenetanshinquinone | MOL007061 | 3.209 |
| IL1B | 3α-hydroxytanshinoneⅡa | MOL007045 | 3.172 |
| IL1B | Diosmetin | MOL002881 | 3.170 |
| IL1B | senkyunone | MOL002151 | 3.157 |
| IL1B | 5-dehydrokarounidiol | MOL007171 | 3.128 |
| IL1B | neokadsuranin | MOL009220 | 3.120 |
| IL1B | Przewaquinone B | MOL007068 | 3.076 |
| IL1B | NSC 122421 | MOL007149 | 3.047 |
| IL1B | α-amyrin | MOL006824 | 3.041 |
| IL1B | przewaquinone f | MOL007071 | 3.026 |
| IL1B | tanshinone Ⅵ | MOL007156 | 3.002 |
| IL1B | miltionone Ⅱ | MOL007120 | 2.988 |
| IL1B | Przewaquinone E | MOL007152 | 2.977 |
| IL1B | Gomisin G | MOL008974 | 2.963 |
| IL1B | formononetin | MOL000392 | 2.962 |
| IL1B | Longikaurin A | MOL004624 | 2.950 |
| IL1B | epidanshenspiroketallactone | MOL007105 | 2.919 |
| IL1B | Bowdichione | MOL002973 | 2.904 |
| IL1B | poriferast-5-en-3beta-ol | MOL001771 | 2.897 |
| IL1B | naringenin | MOL004328 | 2.879 |
| IL1B | salvianolic acid g | MOL007141 | 2.860 |
| IL1B | miltionone Ⅰ | MOL007119 | 2.816 |
| IL1B | przewaquinone c | MOL007069 | 2.798 |
| IL1B | DFV | MOL001792 | 2.791 |
| IL1B | Taraxerol | MOL006554 | 2.778 |
| IL1B | danshenspiroketallactone | MOL007094 | 2.693 |
| IL1B | 1,2,5,6-tetrahydrotanshinone | MOL001601 | 2.663 |
| IL1B | 3-beta-Hydroxymethyllenetanshiquinone | MOL007059 | 2.660 |
| IL1B | formyltanshinone | MOL007058 | 2.602 |
| IL1B | tanshinone iia | MOL007154 | 2.551 |
| IL1B | dihydrotanshinoneⅠ | MOL007101 | 2.542 |
| IL1B | (6S)-6-hydroxy-1-methyl-6-methylol-8,9-dihydro-7H-naphtho[8,7-g]benzofuran-10,11-quinone | MOL007150 | 2.532 |
| IL1B | 1-methyl-8,9-dihydro-7H-naphtho[5,6-g]benzofuran-6,10,11-trione | MOL007127 | 2.529 |
| IL1B | isocryptotanshi-none | MOL007108 | 2.519 |
| IL1B | butin | MOL002975 | 2.503 |
| IL1B | Hydroxygenkwanin | MOL005530 | 2.475 |
| IL1B | dihydrotanshinlactone | MOL007100 | 2.452 |
| IL1B | luteolin | MOL000006 | 2.450 |
| IL1B | miltirone Ⅱ | MOL007123 | 2.444 |
| IL1B | Medicarpin | MOL002565 | 2.410 |
| IL1B | tanshinaldehyde | MOL007079 | 2.387 |
| IL1B | acacetin | MOL001689 | 2.384 |
| IL1B | Dehydrotanshinone II A | MOL002651 | 2.361 |
| IL1B | Isotanshinone II | MOL007111 | 2.266 |
| IL1B | (2R)-7-hydroxy-5-methoxy-2-phenylchroman-4-one | MOL000228 | 2.222 |
| IL1B | Mairin | MOL000211 | 1.964 |
| IL1B | schisanlactone E | MOL009224 | 1.793 |
| TLR2 | Linolenic acid ethyl ester | MOL007179 | 11.070 |
| TLR2 | Supraene | MOL001506 | 10.792 |
| TLR2 | 1-Monolinolein | MOL002464 | 10.238 |
| TLR2 | isomucronulatol-7,2'-di-O-glucosiole | MOL000439 | 9.716 |
| TLR2 | PGA(sup 1) | MOL007650 | 9.629 |
| TLR2 | senkyunone | MOL002151 | 9.611 |
| TLR2 | Prostaglandin B1 | MOL007651 | 9.506 |
| TLR2 | Sitosteryl acetate | MOL001973 | 9.403 |
| TLR2 | Mandenol | MOL001494 | 9.198 |
| TLR2 | FA | MOL000433 | 9.140 |
| TLR2 | 9,10-dimethoxypterocarpan-3-O-β-D-glucoside | MOL000379 | 9.006 |
| TLR2 | vitamin-e | MOL007180 | 8.213 |
| TLR2 | poriferast-5-en-3beta-ol | MOL001771 | 8.163 |
| TLR2 | (2R)-3-(3,4-dihydroxyphenyl)-2-[(Z)-3-(3,4-dihydroxyphenyl)acryloyl]oxy-propionic acid | MOL007132 | 7.873 |
| TLR2 | Daidzein-4,7-diglucoside | MOL003629 | 7.761 |
| TLR2 | neokadsuranic acid B | MOL009218 | 7.742 |
| TLR2 | 6-o-syringyl-8-o-acetyl shanzhiside methyl ester | MOL007051 | 7.723 |
| TLR2 | neokadsuranic acid C | MOL009219 | 7.649 |
| TLR2 | (3S,8S,9S,10R,13R,14S,17R)-10,13-dimethyl-17-[(2R,5S)-5-propan-2-yloctan-2-yl]-2,3,4,7,8,9,11,12,14,15,16,17-dodecahydro-1H-cyclopenta[a]phenanthren-3-ol | MOL000033 | 7.637 |
| TLR2 | salvianolic acid j | MOL007142 | 7.585 |
| TLR2 | changnanic acid | MOL009200 | 7.580 |
| TLR2 | Schottenol | MOL006756 | 7.564 |
| TLR2 | Poriferasterol | MOL001659 | 7.553 |
| TLR2 | Spinasterol | MOL004355 | 7.283 |
| TLR2 | Linarin | MOL001790 | 7.132 |
| TLR2 | sitosterol | MOL000359 | 7.028 |
| TLR2 | (3R)-3-(2,3-dihydroxy-4-methoxyphenyl)chroman-7,8-diol | MOL002941 | 7.015 |
| TLR2 | Baicalin | MOL002776 | 7.012 |
| TLR2 | Deoxyharringtonine | MOL005317 | 6.977 |
| TLR2 | Bifendate | MOL000387 | 6.975 |
| TLR2 | beta-sitosterol | MOL000358 | 6.793 |
| TLR2 | (Z)-3-(4-hydroxy-3-methoxy-phenyl)-N-[2-(4-hydroxyphenyl)ethyl]acrylamide | MOL000483 | 6.670 |
| TLR2 | 2-(4-hydroxy-3-methoxyphenyl)-5-(3-hydroxypropyl)-7-methoxy-3-benzofurancarboxaldehyde | MOL007050 | 6.531 |
| TLR2 | (Z)-3-[2-[(E)-2-(3,4-dihydroxyphenyl)vinyl]-3,4-dihydroxy-phenyl]acrylic acid | MOL007140 | 6.389 |
| TLR2 | n-coumaroyltyramine | MOL000332 | 6.357 |
| TLR2 | wallichilide | MOL002157 | 6.332 |
| TLR2 | Perlolyrine | MOL002140 | 6.315 |
| TLR2 | 5'-hydroxyiso-muronulatol-2',5'-di-O-glucoside | MOL000374 | 6.286 |
| TLR2 | 3-(2-hydroxy-3,4-dimethoxyphenyl)-2H-chromen-7-ol | MOL002997 | 6.222 |
| TLR2 | isoduartin | MOL002985 | 6.191 |
| TLR2 | neokadsuranin | MOL009220 | 6.169 |
| TLR2 | tanshinone Ⅵ | MOL007156 | 6.076 |
| TLR2 | neocryptotanshinone | MOL007125 | 6.075 |
| TLR2 | 5,6-dihydroxy-7-isopropyl-1,1-dimethyl-2,3-dihydrophenanthren-4-one | MOL007036 | 6.060 |
| TLR2 | Przewaquinone E | MOL007152 | 6.042 |
| TLR2 | 3'-Hydroxymelanettin | MOL002958 | 6.016 |
| TLR2 | Salvilenone | MOL007085 | 5.978 |
| TLR2 | salvilenone Ⅰ | MOL007143 | 5.934 |
| TLR2 | isoflavanone | MOL000398 | 5.906 |
| TLR2 | Tanshindiol B | MOL007151 | 5.855 |
| TLR2 | 7-O-methylisomucronulatol | MOL000378 | 5.843 |
| TLR2 | (6aR,11aR)-3,9-dimethoxy-6a,11a-dihydro-6H-benzofurano[3,2-c]chromene-4,10-diol | MOL002991 | 5.840 |
| TLR2 | przewaquinone f | MOL007071 | 5.826 |
| TLR2 | 7-hydroxy-4'-methoxy-2',5'-dioxo-4-[(3R)-2',7-dihydroxy-4'-methoxyisoflavan-5'-yl]isoflavane | MOL002967 | 5.818 |
| TLR2 | prolithospermic acid | MOL007130 | 5.809 |
| TLR2 | (E)-3-[2-(3,4-dihydroxyphenyl)-7-hydroxy-benzofuran-4-yl]acrylic acid | MOL007048 | 5.766 |
| TLR2 | Przewaquinone B | MOL007068 | 5.728 |
| TLR2 | coumaroyltyramine | MOL000631 | 5.723 |
| TLR2 | kadsulactone | MOL009211 | 5.718 |
| TLR2 | neokadsuranic acid A | MOL009217 | 5.706 |
| TLR2 | Jaranol | MOL000239 | 5.695 |
| TLR2 | dan-shexinkum d | MOL007093 | 5.632 |
| TLR2 | Hydroxygenkwanin | MOL005530 | 5.630 |
| TLR2 | Duartin | MOL002981 | 5.610 |
| TLR2 | Myricanone | MOL002135 | 5.583 |
| TLR2 | hederagenin | MOL000296 | 5.580 |
| TLR2 | Vestitone | MOL003001 | 5.572 |
| TLR2 | odoricarpin | MOL002996 | 5.568 |
| TLR2 | miltionone Ⅱ | MOL007120 | 5.568 |
| TLR2 | (-)-Vestitol | MOL002961 | 5.554 |
| TLR2 | 3-beta-Hydroxymethyllenetanshiquinone | MOL007059 | 5.500 |
| TLR2 | 4',5',7-trimethyl-3-methoxyflavone | MOL002963 | 5.493 |
| TLR2 | luteolin | MOL000006 | 5.491 |
| TLR2 | (3R)-3-(2,3-dihydroxy-4-methoxyphenyl)-7-hydroxychroman-4-one | MOL002940 | 5.397 |
| TLR2 | 2-isopropyl-8-methylphenanthrene-3,4-dione | MOL007041 | 5.382 |
| TLR2 | acacetin | MOL001689 | 5.381 |
| TLR2 | isocryptotanshi-none | MOL007108 | 5.343 |
| TLR2 | (2R)-7-hydroxy-5-methoxy-2-phenylchroman-4-one | MOL000228 | 5.302 |
| TLR2 | digallate | MOL000569 | 5.284 |
| TLR2 | 3,9-di-O-methylnissolin | MOL000371 | 5.246 |
| TLR2 | Hesperetin | MOL002341 | 5.191 |
| TLR2 | Sativanone | MOL002999 | 5.160 |
| TLR2 | (6aR,11aR)-3,9,10-trimethoxy-6a,11a-dihydro-6H-benzofurano[3,2-c]chromen-4-ol | MOL002990 | 5.159 |
| TLR2 | kaempferol | MOL000422 | 5.153 |
| TLR2 | Stevein | MOL003000 | 5.143 |
| TLR2 | Danshenol B | MOL007081 | 5.143 |
| TLR2 | violanone | MOL003002 | 5.143 |
| TLR2 | C09092 | MOL007107 | 5.134 |
| TLR2 | (6S)-6-hydroxy-1-methyl-6-methylol-8,9-dihydro-7H-naphtho[8,7-g]benzofuran-10,11-quinone | MOL007150 | 5.124 |
| TLR2 | formyltanshinone | MOL007058 | 5.114 |
| TLR2 | (3S)-7-hydroxy-3-(2,3,4-trimethoxyphenyl)chroman-4-one | MOL002962 | 5.109 |
| TLR2 | butin | MOL002975 | 5.076 |
| TLR2 | Bowdichione | MOL002973 | 5.074 |
| TLR2 | Dehydrotanshinone II A | MOL002651 | 5.056 |
| TLR2 | (2R)-5,7-dihydroxy-2-(4-hydroxyphenyl)chroman-4-one | MOL001040 | 5.021 |
| TLR2 | 4-methylenemiltirone | MOL007049 | 5.012 |
| TLR2 | naringenin | MOL004328 | 5.009 |
| TLR2 | 3'-Methoxydaidzein | MOL002959 | 5.002 |
| TLR2 | deoxyneocryptotanshinone | MOL007098 | 4.984 |
| TLR2 | DFV | MOL001792 | 4.945 |
| TLR2 | Danshenol A | MOL007082 | 4.914 |
| TLR2 | (6aR,11aR)-9,10-dimethoxy-6a,11a-dihydro-6H-benzofurano[3,2-c]chromen-3-ol | MOL000380 | 4.885 |
| TLR2 | schisanlactone E | MOL009224 | 4.854 |
| TLR2 | Xenognosin B | MOL003003 | 4.828 |
| TLR2 | (3R)-7,2',3'-trihydroxy-4-methoxyisoflavan | MOL002950 | 4.772 |
| TLR2 | manool | MOL007115 | 4.762 |
| TLR2 | dihydrotanshinlactone | MOL007100 | 4.735 |
| TLR2 | 1,7-Dihydroxy-3,9-dimethoxy pterocarpene | MOL000442 | 4.704 |
| TLR2 | salvianolic acid g | MOL007141 | 4.684 |
| TLR2 | danshenspiroketallactone | MOL007094 | 4.679 |
| TLR2 | (6S)-6-(hydroxymethyl)-1,6-dimethyl-8,9-dihydro-7H-naphtho[8,7-g]benzofuran-10,11-dione | MOL007155 | 4.673 |
| TLR2 | quercetin | MOL000098 | 4.658 |
| TLR2 | isoimperatorin | MOL001942 | 4.652 |
| TLR2 | (6S,7R)-6,7-dihydroxy-1,6-dimethyl-8,9-dihydro-7H-naphtho[8,7-g]benzofuran-10,11-dione | MOL007070 | 4.638 |
| TLR2 | Diosmetin | MOL002881 | 4.626 |
| TLR2 | (3R)-3-(2-hydroxy-3,4-dimethoxyphenyl)chroman-7-ol | MOL000438 | 4.618 |
| TLR2 | sugiol | MOL002222 | 4.608 |
| TLR2 | epidanshenspiroketallactone | MOL007105 | 4.588 |
| TLR2 | Calycosin | MOL000417 | 4.559 |
| TLR2 | Miltirone | MOL007122 | 4.558 |
| TLR2 | macrostemonoside e_qt | MOL007640 | 4.527 |
| TLR2 | isorhamnetin | MOL000354 | 4.513 |
| TLR2 | NSC 122421 | MOL007149 | 4.454 |
| TLR2 | przewalskin b | MOL007064 | 4.453 |
| TLR2 | Isotanshinone II | MOL007111 | 4.388 |
| TLR2 | Gomisin-A | MOL008968 | 4.361 |
| TLR2 | Medicarpin | MOL002565 | 4.352 |
| TLR2 | przewaquinone c | MOL007069 | 4.351 |
| TLR2 | 9-O-Methylcoumestrol | MOL002957 | 4.307 |
| TLR2 | Mairin | MOL000211 | 4.279 |
| TLR2 | (3R)-5'-Methoxyvestitol | MOL002939 | 4.260 |
| TLR2 | dihydrotanshinoneⅠ | MOL007101 | 4.250 |
| TLR2 | miltionone Ⅰ | MOL007119 | 4.246 |
| TLR2 | tanshinone iia | MOL007154 | 4.164 |
| TLR2 | formononetin | MOL000392 | 4.139 |
| TLR2 | 4-Hydroxyhomopterocarpin | MOL002989 | 4.112 |
| TLR2 | neocryptotanshinone ii | MOL007124 | 4.110 |
| TLR2 | Eriodyctiol (flavanone) | MOL002914 | 4.077 |
| TLR2 | Methylenetanshinquinone | MOL007061 | 4.068 |
| TLR2 | kadsulignan B | MOL009213 | 4.004 |
| TLR2 | tanshinaldehyde | MOL007079 | 3.927 |
| TLR2 | (3R)-4'-Methoxy-2',3,7-trihydroxyisoflavanone | MOL002938 | 3.888 |
| TLR2 | salviolone | MOL007145 | 3.884 |
| TLR2 | 1-methyl-8,9-dihydro-7H-naphtho[5,6-g]benzofuran-6,10,11-trione | MOL007127 | 3.859 |
| TLR2 | 1,2,5,6-tetrahydrotanshinone | MOL001601 | 3.692 |
| TLR2 | schizandronic acid | MOL009229 | 3.532 |
| TLR2 | miltirone Ⅱ | MOL007123 | 3.477 |
| TLR2 | 3α-hydroxytanshinoneⅡa | MOL007045 | 3.439 |
| TLR2 | przewalskin a | MOL007063 | 3.075 |
| TLR2 | sclareol | MOL007077 | 2.977 |
| TLR2 | microstegiol | MOL007118 | 2.689 |
| TLR2 | schisanlactone A | MOL009222 | 2.286 |
| TLR2 | Gomisin R | MOL008978 | 2.064 |
| TLR2 | miltipolone | MOL007121 | 1.588 |
| TLR2 | Wuweizisu C | MOL008992 | 1.280 |
| TLR2 | Longikaurin A | MOL004624 | 1.198 |
| TLR2 | Schizandrer B | MOL008957 | 0.764 |
| TLR2 | α-amyrin | MOL006824 | 0.762 |
| TLR2 | Taraxerol | MOL006554 | 0.345 |
| TLR2 | (3R,4R)-3',7-dihydroxy-2',4'-dimethoxy-4-[(2S)-4',5,7-trihydroxyflavanone-6-yl]isoflavan | MOL002982 | 0.086 |
| TLR2 | angusifolin B | MOL009235 | -0.159 |
| TLR2 | 7-oxo-dihydrokaro-unidiol | MOL007172 | -0.176 |
| TLR2 | Interiotherin B | MOL009199 | -0.361 |
| TLR2 | Kadsulignan C | MOL009210 | -0.467 |
| TLR2 | Gomisin G | MOL008974 | -0.554 |
| TLR2 | 5-dehydrokarounidiol | MOL007171 | -0.818 |
| TLR2 | 10α-cucurbita-5,24-diene-3β-ol | MOL007165 | -1.265 |
| TLR2 | karounidiol 3-o-benzoate | MOL007175 | -6.717 |
| TLR2 | Angeloylgomisin O | MOL008956 | -7.514 |
| TXNRD1 | Mandenol | MOL001494 | 5.248 |
| TXNRD1 | Linolenic acid ethyl ester | MOL007179 | 5.007 |
| TXNRD1 | senkyunone | MOL002151 | 4.975 |
| TXNRD1 | (Z)-3-(4-hydroxy-3-methoxy-phenyl)-N-[2-(4-hydroxyphenyl)ethyl]acrylamide | MOL000483 | 4.799 |
| TXNRD1 | 1-Monolinolein | MOL002464 | 4.645 |
| TXNRD1 | 3'-Hydroxymelanettin | MOL002958 | 4.630 |
| TXNRD1 | wallichilide | MOL002157 | 4.395 |
| TXNRD1 | Prostaglandin B1 | MOL007651 | 4.341 |
| TXNRD1 | neokadsuranic acid B | MOL009218 | 4.292 |
| TXNRD1 | Sitosteryl acetate | MOL001973 | 4.195 |
| TXNRD1 | Spinasterol | MOL004355 | 4.181 |
| TXNRD1 | Supraene | MOL001506 | 4.108 |
| TXNRD1 | isoduartin | MOL002985 | 4.023 |
| TXNRD1 | neokadsuranic acid A | MOL009217 | 3.981 |
| TXNRD1 | coumaroyltyramine | MOL000631 | 3.950 |
| TXNRD1 | 7-O-methylisomucronulatol | MOL000378 | 3.838 |
| TXNRD1 | 3-(2-hydroxy-3,4-dimethoxyphenyl)-2H-chromen-7-ol | MOL002997 | 3.792 |
| TXNRD1 | Schottenol | MOL006756 | 3.748 |
| TXNRD1 | Danshenol B | MOL007081 | 3.569 |
| TXNRD1 | (2R)-3-(3,4-dihydroxyphenyl)-2-[(Z)-3-(3,4-dihydroxyphenyl)acryloyl]oxy-propionic acid | MOL007132 | 3.518 |
| TXNRD1 | Eriodyctiol (flavanone) | MOL002914 | 3.481 |
| TXNRD1 | Danshenol A | MOL007082 | 3.472 |
| TXNRD1 | neokadsuranic acid C | MOL009219 | 3.370 |
| TXNRD1 | schizandronic acid | MOL009229 | 3.331 |
| TXNRD1 | n-coumaroyltyramine | MOL000332 | 3.318 |
| TXNRD1 | isomucronulatol-7,2'-di-O-glucosiole | MOL000439 | 3.225 |
| TXNRD1 | (3S,8S,9S,10R,13R,14S,17R)-10,13-dimethyl-17-[(2R,5S)-5-propan-2-yloctan-2-yl]-2,3,4,7,8,9,11,12,14,15,16,17-dodecahydro-1H-cyclopenta[a]phenanthren-3-ol | MOL000033 | 3.219 |
| TXNRD1 | Wuweizisu C | MOL008992 | 3.158 |
| TXNRD1 | (3R)-3-(2-hydroxy-3,4-dimethoxyphenyl)chroman-7-ol | MOL000438 | 3.157 |
| TXNRD1 | (3R)-3-(2,3-dihydroxy-4-methoxyphenyl)chroman-7,8-diol | MOL002941 | 3.066 |
| TXNRD1 | macrostemonoside e_qt | MOL007640 | 3.006 |
| TXNRD1 | Bifendate | MOL000387 | 3.000 |
| TXNRD1 | (6S)-6-(hydroxymethyl)-1,6-dimethyl-8,9-dihydro-7H-naphtho[8,7-g]benzofuran-10,11-dione | MOL007155 | 2.997 |
| TXNRD1 | kadsulactone | MOL009211 | 2.973 |
| TXNRD1 | (3S)-7-hydroxy-3-(2,3,4-trimethoxyphenyl)chroman-4-one | MOL002962 | 2.951 |
| TXNRD1 | changnanic acid | MOL009200 | 2.857 |
| TXNRD1 | Gomisin R | MOL008978 | 2.852 |
| TXNRD1 | beta-sitosterol | MOL000358 | 2.789 |
| TXNRD1 | 10α-cucurbita-5,24-diene-3β-ol | MOL007165 | 2.756 |
| TXNRD1 | (2R)-7-hydroxy-5-methoxy-2-phenylchroman-4-one | MOL000228 | 2.740 |
| TXNRD1 | (-)-Vestitol | MOL002961 | 2.716 |
| TXNRD1 | Deoxyharringtonine | MOL005317 | 2.698 |
| TXNRD1 | 4-Hydroxyhomopterocarpin | MOL002989 | 2.666 |
| TXNRD1 | dihydrotanshinlactone | MOL007100 | 2.665 |
| TXNRD1 | Sativanone | MOL002999 | 2.643 |
| TXNRD1 | (3R)-3-(2,3-dihydroxy-4-methoxyphenyl)-7-hydroxychroman-4-one | MOL002940 | 2.601 |
| TXNRD1 | poriferast-5-en-3beta-ol | MOL001771 | 2.542 |
| TXNRD1 | 2-(4-hydroxy-3-methoxyphenyl)-5-(3-hydroxypropyl)-7-methoxy-3-benzofurancarboxaldehyde | MOL007050 | 2.535 |
| TXNRD1 | (6aR,11aR)-3,9,10-trimethoxy-6a,11a-dihydro-6H-benzofurano[3,2-c]chromen-4-ol | MOL002990 | 2.529 |
| TXNRD1 | przewaquinone f | MOL007071 | 2.528 |
| TXNRD1 | (3R)-5'-Methoxyvestitol | MOL002939 | 2.506 |
| TXNRD1 | Xenognosin B | MOL003003 | 2.497 |
| TXNRD1 | isoimperatorin | MOL001942 | 2.491 |
| TXNRD1 | Baicalin | MOL002776 | 2.476 |
| TXNRD1 | naringenin | MOL004328 | 2.475 |
| TXNRD1 | 4',5',7-trimethyl-3-methoxyflavone | MOL002963 | 2.470 |
| TXNRD1 | schisanlactone A | MOL009222 | 2.460 |
| TXNRD1 | prolithospermic acid | MOL007130 | 2.439 |
| TXNRD1 | neocryptotanshinone | MOL007125 | 2.411 |
| TXNRD1 | angusifolin B | MOL009235 | 2.398 |
| TXNRD1 | vitamin-e | MOL007180 | 2.359 |
| TXNRD1 | digallate | MOL000569 | 2.328 |
| TXNRD1 | Salvilenone | MOL007085 | 2.322 |
| TXNRD1 | deoxyneocryptotanshinone | MOL007098 | 2.321 |
| TXNRD1 | Vestitone | MOL003001 | 2.318 |
| TXNRD1 | odoricarpin | MOL002996 | 2.308 |
| TXNRD1 | hederagenin | MOL000296 | 2.296 |
| TXNRD1 | Isotanshinone II | MOL007111 | 2.276 |
| TXNRD1 | violanone | MOL003002 | 2.270 |
| TXNRD1 | 7-hydroxy-4'-methoxy-2',5'-dioxo-4-[(3R)-2',7-dihydroxy-4'-methoxyisoflavan-5'-yl]isoflavane | MOL002967 | 2.212 |
| TXNRD1 | Duartin | MOL002981 | 2.211 |
| TXNRD1 | sitosterol | MOL000359 | 2.167 |
| TXNRD1 | quercetin | MOL000098 | 2.154 |
| TXNRD1 | dihydrotanshinoneⅠ | MOL007101 | 2.143 |
| TXNRD1 | FA | MOL000433 | 2.105 |
| TXNRD1 | Gomisin G | MOL008974 | 2.064 |
| TXNRD1 | przewaquinone c | MOL007069 | 2.034 |
| TXNRD1 | 9,10-dimethoxypterocarpan-3-O-β-D-glucoside | MOL000379 | 2.011 |
| TXNRD1 | Methylenetanshinquinone | MOL007061 | 2.006 |
| TXNRD1 | 3α-hydroxytanshinoneⅡa | MOL007045 | 1.964 |
| TXNRD1 | Kadsulignan C | MOL009210 | 1.953 |
| TXNRD1 | sugiol | MOL002222 | 1.932 |
| TXNRD1 | formononetin | MOL000392 | 1.926 |
| TXNRD1 | kadsulignan B | MOL009213 | 1.922 |
| TXNRD1 | manool | MOL007115 | 1.921 |
| TXNRD1 | Dehydrotanshinone II A | MOL002651 | 1.901 |
| TXNRD1 | (3R)-7,2',3'-trihydroxy-4-methoxyisoflavan | MOL002950 | 1.892 |
| TXNRD1 | α-amyrin | MOL006824 | 1.892 |
| TXNRD1 | salviolone | MOL007145 | 1.871 |
| TXNRD1 | 1,7-Dihydroxy-3,9-dimethoxy pterocarpene | MOL000442 | 1.860 |
| TXNRD1 | (6aR,11aR)-3,9-dimethoxy-6a,11a-dihydro-6H-benzofurano[3,2-c]chromene-4,10-diol | MOL002991 | 1.856 |
| TXNRD1 | 1,2,5,6-tetrahydrotanshinone | MOL001601 | 1.843 |
| TXNRD1 | 5'-hydroxyiso-muronulatol-2',5'-di-O-glucoside | MOL000374 | 1.827 |
| TXNRD1 | Hesperetin | MOL002341 | 1.820 |
| TXNRD1 | tanshinone Ⅵ | MOL007156 | 1.816 |
| TXNRD1 | przewalskin a | MOL007063 | 1.812 |
| TXNRD1 | C09092 | MOL007107 | 1.794 |
| TXNRD1 | Przewaquinone E | MOL007152 | 1.790 |
| TXNRD1 | formyltanshinone | MOL007058 | 1.776 |
| TXNRD1 | Calycosin | MOL000417 | 1.753 |
| TXNRD1 | 3-beta-Hydroxymethyllenetanshiquinone | MOL007059 | 1.736 |
| TXNRD1 | dan-shexinkum d | MOL007093 | 1.721 |
| TXNRD1 | Myricanone | MOL002135 | 1.715 |
| TXNRD1 | 3'-Methoxydaidzein | MOL002959 | 1.710 |
| TXNRD1 | (E)-3-[2-(3,4-dihydroxyphenyl)-7-hydroxy-benzofuran-4-yl]acrylic acid | MOL007048 | 1.699 |
| TXNRD1 | PGA(sup 1) | MOL007650 | 1.689 |
| TXNRD1 | miltipolone | MOL007121 | 1.684 |
| TXNRD1 | miltionone Ⅱ | MOL007120 | 1.642 |
| TXNRD1 | sclareol | MOL007077 | 1.633 |
| TXNRD1 | tanshinaldehyde | MOL007079 | 1.628 |
| TXNRD1 | Przewaquinone B | MOL007068 | 1.627 |
| TXNRD1 | epidanshenspiroketallactone | MOL007105 | 1.626 |
| TXNRD1 | Stevein | MOL003000 | 1.625 |
| TXNRD1 | (6S)-6-hydroxy-1-methyl-6-methylol-8,9-dihydro-7H-naphtho[8,7-g]benzofuran-10,11-quinone | MOL007150 | 1.610 |
| TXNRD1 | neokadsuranin | MOL009220 | 1.586 |
| TXNRD1 | miltirone Ⅱ | MOL007123 | 1.565 |
| TXNRD1 | Gomisin-A | MOL008968 | 1.564 |
| TXNRD1 | NSC 122421 | MOL007149 | 1.548 |
| TXNRD1 | isocryptotanshi-none | MOL007108 | 1.526 |
| TXNRD1 | (6S,7R)-6,7-dihydroxy-1,6-dimethyl-8,9-dihydro-7H-naphtho[8,7-g]benzofuran-10,11-dione | MOL007070 | 1.515 |
| TXNRD1 | 1-methyl-8,9-dihydro-7H-naphtho[5,6-g]benzofuran-6,10,11-trione | MOL007127 | 1.504 |
| TXNRD1 | Angeloylgomisin O | MOL008956 | 1.466 |
| TXNRD1 | 7-oxo-dihydrokaro-unidiol | MOL007172 | 1.458 |
| TXNRD1 | Linarin | MOL001790 | 1.442 |
| TXNRD1 | isoflavanone | MOL000398 | 1.347 |
| TXNRD1 | 9-O-Methylcoumestrol | MOL002957 | 1.321 |
| TXNRD1 | Tanshindiol B | MOL007151 | 1.314 |
| TXNRD1 | 5-dehydrokarounidiol | MOL007171 | 1.313 |
| TXNRD1 | przewalskin b | MOL007064 | 1.247 |
| TXNRD1 | Perlolyrine | MOL002140 | 1.190 |
| TXNRD1 | neocryptotanshinone ii | MOL007124 | 1.182 |
| TXNRD1 | isorhamnetin | MOL000354 | 1.162 |
| TXNRD1 | Jaranol | MOL000239 | 1.139 |
| TXNRD1 | Interiotherin B | MOL009199 | 1.132 |
| TXNRD1 | 2-isopropyl-8-methylphenanthrene-3,4-dione | MOL007041 | 1.128 |
| TXNRD1 | salvilenone Ⅰ | MOL007143 | 1.127 |
| TXNRD1 | DFV | MOL001792 | 1.060 |
| TXNRD1 | microstegiol | MOL007118 | 1.054 |
| TXNRD1 | Medicarpin | MOL002565 | 1.046 |
| TXNRD1 | tanshinone iia | MOL007154 | 1.045 |
| TXNRD1 | Taraxerol | MOL006554 | 1.039 |
| TXNRD1 | (6aR,11aR)-9,10-dimethoxy-6a,11a-dihydro-6H-benzofurano[3,2-c]chromen-3-ol | MOL000380 | 1.032 |
| TXNRD1 | acacetin | MOL001689 | 1.029 |
| TXNRD1 | (Z)-3-[2-[(E)-2-(3,4-dihydroxyphenyl)vinyl]-3,4-dihydroxy-phenyl]acrylic acid | MOL007140 | 0.951 |
| TXNRD1 | miltionone Ⅰ | MOL007119 | 0.937 |
| TXNRD1 | Schizandrer B | MOL008957 | 0.937 |
| TXNRD1 | 3,9-di-O-methylnissolin | MOL000371 | 0.900 |
| TXNRD1 | Bowdichione | MOL002973 | 0.880 |
| TXNRD1 | karounidiol 3-o-benzoate | MOL007175 | 0.879 |
| TXNRD1 | 4-methylenemiltirone | MOL007049 | 0.863 |
| TXNRD1 | butin | MOL002975 | 0.826 |
| TXNRD1 | danshenspiroketallactone | MOL007094 | 0.816 |
| TXNRD1 | (3R)-4'-Methoxy-2',3,7-trihydroxyisoflavanone | MOL002938 | 0.810 |
| TXNRD1 | salvianolic acid j | MOL007142 | 0.795 |
| TXNRD1 | Miltirone | MOL007122 | 0.773 |
| TXNRD1 | (3R,4R)-3',7-dihydroxy-2',4'-dimethoxy-4-[(2S)-4',5,7-trihydroxyflavanone-6-yl]isoflavan | MOL002982 | 0.741 |
| TXNRD1 | 6-o-syringyl-8-o-acetyl shanzhiside methyl ester | MOL007051 | 0.709 |
| TXNRD1 | luteolin | MOL000006 | 0.708 |
| TXNRD1 | Diosmetin | MOL002881 | 0.614 |
| TXNRD1 | Hydroxygenkwanin | MOL005530 | 0.602 |
| TXNRD1 | schisanlactone E | MOL009224 | 0.583 |
| TXNRD1 | 5,6-dihydroxy-7-isopropyl-1,1-dimethyl-2,3-dihydrophenanthren-4-one | MOL007036 | 0.544 |
| TXNRD1 | salvianolic acid g | MOL007141 | 0.532 |
| TXNRD1 | Mairin | MOL000211 | 0.384 |
| TXNRD1 | Poriferasterol | MOL001659 | 0.350 |
| TXNRD1 | kaempferol | MOL000422 | 0.244 |
| TXNRD1 | Longikaurin A | MOL004624 | 0.049 |
| TXNRD1 | (2R)-5,7-dihydroxy-2-(4-hydroxyphenyl)chroman-4-one | MOL001040 | 0.028 |
| TXNRD1 | Daidzein-4,7-diglucoside | MOL003629 | -0.411 |
